# Genome-wide identification, evolutionary and expression analysis of the cyclin-dependent kinase gene family in peanut

**DOI:** 10.1186/s12870-023-04045-w

**Published:** 2023-01-19

**Authors:** Gokul Babu S, Deependra Singh Gohil, Swarup Roy Choudhury

**Affiliations:** Department of Biology, Indian Institute of Science Education and Research (IISER) Tirupati, Tirupati, Andhra Pradesh 517507 India

**Keywords:** Abiotic stress, Correlation, Cyclin-dependent kinases, Gene duplication, Gene expression, Motif, Peanut, Promoter

## Abstract

**Background:**

Cyclin-dependent kinases (CDKs) are a predominant group of serine/threonine protein kinases that have multi-faceted functions in eukaryotes. The plant CDK members have well-known roles in cell cycle progression, transcriptional regulation, DNA repair, abiotic stress and defense responses, making them promising targets for developing stress adaptable high-yielding crops. There is relatively sparse information available on the CDK family genes of cultivated oilseed crop peanut and its diploid progenitors.

**Results:**

We have identified 52 putative cyclin-dependent kinases (*CDKs*) and CDK-like (*CDKL*s) genes in *Arachis hypogaea* (cultivated peanut) and total 26 genes in each diploid parent of cultivated peanut (*Arachis duranensis* and *Arachis ipaensis*). Both *CDK* and *CDKL* genes were classified into eight groups based on their cyclin binding motifs and their phylogenetic relationship with *Arabidopsis* counterparts. Genes in the same subgroup displayed similar exon–intron structure and conserved motifs. Further, gene duplication analysis suggested that segmental duplication events played major roles in the expansion and evolution of *CDK* and *CDKL* genes in cultivated peanuts*. *Identification of diverse cis-acting response elements in *CDK* and *CDKL* genes promoter indicated their potential fundamental roles in multiple biological processes. Various gene expression patterns of *CDKs* and *CDKLs* in different peanut tissues suggested their involvement during growth and development. In addition, qRT-PCR analysis demonstrated that most representing *CDK* and *CDKL* gene family members were significantly down-regulated under ABA, PEG and mannitol treatments.

**Conclusions:**

Genome-wide analysis offers a comprehensive understanding of the classification, evolution, gene structure, and gene expression profiles of *CDK* and *CDKL* genes in cultivated peanut and their diploid progenitors. Additionally, it also provides cell cycle regulatory gene resources for further functional characterization to enhance growth, development and abiotic stress tolerance.

**Supplementary Information:**

The online version contains supplementary material available at 10.1186/s12870-023-04045-w.

## Background

The cell cycle comprises precisely orchestrated steps through which a cell duplicates its genetic material, which leads to the formation of new cells. Developing new daughter cells through cell division is a key driving force regulating growth in eukaryotes. To ensure genetic integrity, cell division is precisely coordinated by intrinsic and extrinsic cues, including cell cycle regulators, which subsequently control metabolism and growth. Among cell cycle regulators, an evolutionarily conserved protein kinase family called cyclin-dependent kinases (CDKs) are the major controllers [1]. The cell cycle progression primarily depends on the interaction of CDKs with their cyclin partners; and this association promptly endorses the oscillation of CDKs on its targets during the process [2].

CDKs belong to the CMGC group of protein kinase, which also includes MAPK and GSK-3. CDKs are also members of serine/threonine protein kinases with an amino-terminal lobe encompassing beta-sheets and a carboxy-terminal lobe which mostly constitutes of α-helices [3]. A glycine-rich inhibitory element (G-loop) and a cyclin binding evolutionary conserved 16 amino acid sequence (EGVPSTAI-REISLLKE) motif called PSTAIRE remain in the N-lobe (PSTAIRE motif in case of CDKA). The C-lobe contains an extended activation segment comprising a phosphorylation-sensitive T-loop in between both DFG and APE motif required for accelerating enzymatic activity by unlocking the catalytic pocket [3, 4]. Although substrate binding and stabilization of the interaction between kinase-substrate are stringently operated by C-lobe, the kinase activity can be inhibited by phosphorylation of threonine residues at G-loop in the N-lobe [5]. The CDKs are functionally highly conserved, but their numbers vary among different organisms. For instance, the mammalian genome encodes nearly 20 CDKs, whereas in yeast there are only six [6]. Despite its role in cell cycle progression, CDKs are also involved in DNA repair, transcriptional regulation, and proteolytic degradation mechanisms in mammals [7, 8].

During the last few decades, considerable research on CDK-mediated plant cell proliferation has established that the structural and functional properties of CDKs are mostly similar among plants and animals. Although common cell cycle tools are operational in different organisms, the absence of certain animal and yeast CDK counterparts or presence of new CDK subunits in plants is probably essential for cytoskeleton organization and cytokinesis, which are unique to plant cell cycle [9]. Based on cyclin binding motifs, total of 29 CDK genes are subdivided into eight groups in the model plant Arabidopsis, and these CDKs influence core cell-cycle regulation in plants. In Arabidopsis CDKA;1, homologous to the mammalian Cdk1/Cdk2, contains the conserved PSTAIRE motif and has implicated roles in G1/S-G2/M transitions, retinoblastoma homolog RBR1 control, and meiotic crossovers formation [10, 11]. The plant-specific CDKB group, subdivided into CDKB1 and CDKB2, harbor the PPT(A/T) LRE cyclin binding motif. In addition to their roles in G2/M transitions, Arabidopsis CDKB1 controls homology-dependent repair through substrates like RAD51, whereas CDKB2 maintains the shoot apical meristem integrity [12–14]. The CDKC group that contains PITAIRE cyclin-binding motif are mammalian CDK9 orthologs. Analysis of loss of function mutants of two CDKC members in Arabidopsis, CDKC;1 and CDKC;2 suggested their roles in infection with cauliflower mosaic virus by transcriptional activation of viral genes. *Cdkc;2* mutants show better immunity to the virus, increased cell numbers, and decreased stomatal density essential for drought tolerance [15, 16]. In addition, a canonical MAP kinase (MAPK) cascade-mediated stimulation activates CDKC members in a phosphorylation-dependent manner. Thus, CDKC members can act as downstream targets of multiple immune receptors [17]. CDKC;2 is also a major transcriptional regulator as it phosphorylates the C-terminal domains of RNA polymerase II [18]. CDKE members in Arabidopsis share a SPTAIRE cyclin binding motif and other structural characteristics with mammalian CDK8. Phenotypic analysis revealed that HUA ENHANCER 3 (HEN3) or E-type CDK is involved in leaf cell expansion, cell differentiation and floral meristem cell fate specifications [19]. CDKE also mediates plant defense responses by inducing defensin genes, transcriptional regulation of secondary metabolites and cuticular wax biosynthesis [20]. CDKG members with conserved PLTSLRE motif are homologous to mammalian CDK10/11 [21]. Arabidopsis genome encodes two copies of CDKG, namely CDKG1 and CDKG2. The former regulates alternate splicing of callose synthase 5 and is necessary for pollen wall formation, recombination intermediates stabilization, synapsis, and homology-dependent somatic DNA repair, while the product of CDKG2 appears to be required in salinity stress responses, modulation of flowering time in response to salt stress, alternative splicing of flowering locus M (FLM) and the temperature-dependent alternate splicing of CDKG1 [22–26]. CDK activation requires T-loop domain phosphorylation by the CDK activating kinases (CAKs), and two groups of plant CDKs, CDKD and CDKF, exhibit maximum CDK kinase activity. The CDKD members have N(I/V/F) TALRE cyclin-binding motifs and are associated with H-type cyclins. Arabidopsis has three distinct types of CDKD (CDKD;1 to CDKD;3), within them CDKD;2 and CDKD;3 show CDK kinase activity and regulate basal transcription by phosphorylating the carboxy-terminal domain (CTD) of RNA polymerase II [27]. CDKD;1 exhibits neither CDK nor CTD kinase activity;1 [28]. CDKD;3 dependent CDKA;1 activation plays an important role in the prophase I microtubular organization and cytokinesis pattern determination [29]. CDKF can act as CAK and CAK activating kinase or CAKAK, as it stimulates CTD kinase activity of CDKD;2 and phosphorylates the T-loop of both CDKD;2 and CDKD;3 to maintain their steady-state level, respectively [30, 31]. Cell division, cell elongation and endoreduplication are significantly impaired *cdkf;1–1* mutants suggesting that CDKF significantly affects cell cycle [30]. The last group of CDK kinases is called CDK-like kinases (CDKLs) that contain a (V/I/L)(K/R)FMAREI cyclin-binding motif, and in Arabidopsis fifteen members CDKLs are crucial for phosphorylation of multiple cell cycle regulators [32].

The legume family (Fabaceae or Leguminosae) is a significant flowering plant family consisting of nearly 20,000 species, and more than 85% of them are involved in symbiotic nitrogen fixation [33]. Multiple economically and nutritionally important plants, including cultivated peanut or groundnut, belong to this family. Cultivated peanut (*Arachis hypogaea* L.) is a polyploid legume oilseed crop with a high protein content grown in the tropical and subtropical regions [34]. Presumably, hybridising two diploids (*Arachis duranensis* and *Arachis ipaensis*), followed by chromosomal duplication, resulted in allotetraploid cultivated peanut [35]. CDKs are not only one of the major determinants of plant growth and development; they can also modulate multiple stress and hormonal responses [36]. In addition, information related to CDKs in peanuts is obscure, thus, an extensive study on CDK family members in peanuts is a prerequisite. The availability of a newer version of the peanut genome has provided opportunities to study genome-wide identification of CDKs from groundnut [37]. Here, we have identified CDK family members from cultivated peanut and their progenitors and examined their phylogenetic relationship, gene architecture, conserved motifs, chromosomal localization, promoter elements, whole-genome duplications, syntenic relationship, selection pressures, gene expressions patterns in tissues and their response to drought stress.

## Results

### Genome-wide identification of *CDK* and *CDKL* gene family in wild-type and cultivated peanuts

To identify CDK and CDK like (CDKL) proteins in peanut genome, 29 Arabidopsis CDK and CDKL proteins were used as query to BLAST the reference genomes of several *Arachis species* deposited in the Peanut database (https://peanutbase.org/home). After a similarity sequence comparison, redundant genes were removed. Finally, 52 putative cyclin-dependent genes were identified in *Arachis hypogaea* (cultivated peanut), of which 24 was *CDKs,* and 28 was *CDKLs*. Each diploid parent of cultivated peanut (*Arachis duranensis* and *Arachis ipaensis*) encoded 26 genes, which included 12 *CDKs* and 14 *CDKLs*. Although these genes belong to the CDK family, their sizes and physicochemical properties vary significantly. According to sequence analysis, the full-length open reading frame (ORF) of *CDK* genes in *A. hypogaea* (Ah) ranged from 885 to 2262 bp; with putative protein sequences within the range 294–753 amino acids (aa) and molecular weights (MW) within the range ~ 33.7–84.3 kDa. Besides, the full-length open reading frame (ORF) of *CDKL* genes in *A. hypogaea* ranged from 1542 to 2307 bp; with putative protein sequences within the range 513–768 amino acids (aa) and molecular weights (MW) within the range ~ 57.0–85.8 kDa (Table S1). The range of putative protein sequences of *CDK* and *CDKL* genes in *A. duranensis* (Ad) were 279 to 784 and 511 to 709, respectively. In *A. ipaensis* (Ai), the range of putative protein sequences of *CDK* and *CDKL* genes were 253 to 832 and 544 to 709 (Table S1). Similar to CDK and CDKL proteins in Arabidopsis, most CDKs and CDKLs were predicted to be localized exclusively to the nucleus or cytoplasm or both the nucleus and cytoplasm [38]. Typically, all CDKs of three *Arachis species* were localized in the nucleus, cytoplasm, or both. Near about 75% AhCDKLs, 78% AdCDKLs and 78% AiCDKLs were restricted in nucleus, and, a few of the CDKLs like 7 AhCDKLs, 3 AdCDKLs and 3 AiCDKLs were localized in either mitochondria or in both the mitochondria and nucleus (Table S1).

In *A. hypogaea*, the intron percentages of CDKs and CDKLs ranged from 10.3% to 65.3% and 23.3% to 64%, respectively*. *The *A. duranensis* and *A. ipaensis* intron percentages for CDKs ranged from 13.4% to 63.2% and 11.8% to 65.6%, respectively and for CDKLs this range varied from 25.6% to 60.2% and 17.1% to 63.4%, respectively.

CDK and CDKL proteins can control successive cell cycle phases mostly upon binding of regulatory proteins such as cyclins [39]. Thus, CDK proteins were classified into eight groups based on their cyclin binding motifs in Arabidopsis [32]. Here, CDKs and CDKLs in three different *Arachis species* are also categorized into eight classes as they contain eight specific kinds of cyclin binding motifs. Like other plant CDK proteins, CDKA, CDKB1, CDKB2, CDKC, CDKD, CDKE, CDKG, CDK-like (CDKL) had a conserved PSTAIRE, PPTALRE, PPTTLRE, PITAIRE, N(I/V/F)TALRE, SPTAIRE, PL(T/S)SLRE, (V/I/L)(K/R)FMAREI motifs, respectively (Fig. S1 and Fig. S2). A 20 to 30 residues long activation loop (T-loop) region remained between the conserved DFG motif (Asp-Phe-Gly) and APE motif (Ala-Pro-Glu) in CDKs except CDKF. CDKA, CDKE, CDKD and CDKG members had defined 20 to 30 residue-long activation loop (T-loop) regions, whereas in other CDK group members, a few variations in the conserved motifs were observed. For example, CDKB members had a DLG motif instead of a DFG motif, whereas CDKC members had PPE motif instead of an APE motif. All CDK members except CDKE possessed the conserved phosphorylated threonine residue (Fig. S3, Fig. S4).

### Phylogenetic relationship of *CDK* and *CDKL* gene family between wild-type and cultivated peanuts

**Fig. 1:**
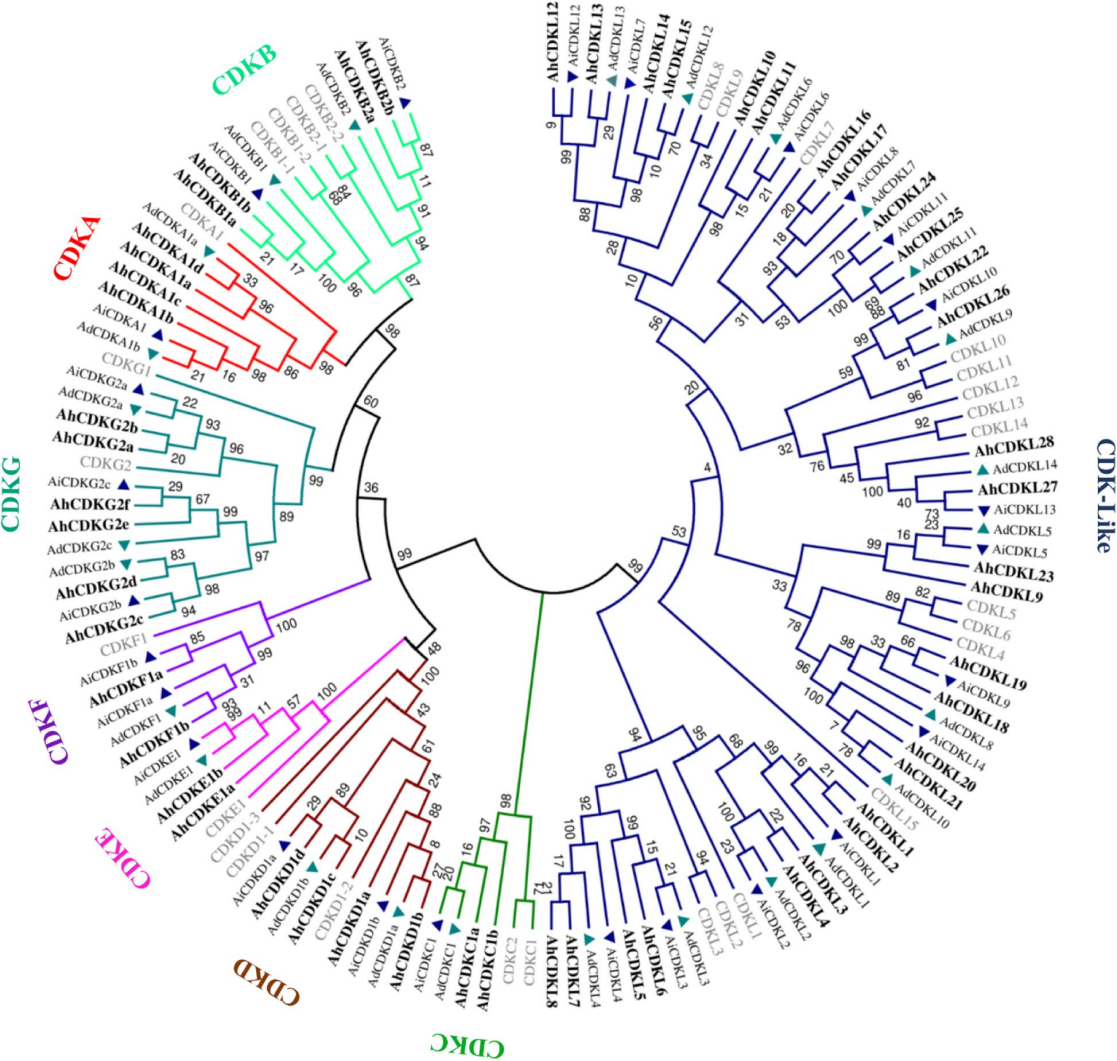
Phylogenetic tree of CDKs and CDKLs from *Arachis hypogaea, Arachis duranensis, Arachis ipaensis* and *Arabidopsis thaliana*. The phylogenetic tree was constructed using MEGA 7.0 by the Maximum-Likelihood method with 1000 bootstrap replications. The tree was classified into 8 different subfamilies (CDKA, CDKB, CDKC, CDKD, CDKE, CDKF, CDKG and CDK-like or CDKL) indicated by different color lines. Names in bold represent CDKs and CDKLs of *Arachis hypogaea*, and the grey colored names show CDKs and CDKLs of Arabidopsis. Upright blue triangles represent CDKs and CDKLs of *Arachis ipaensis*, whereas the inverted green triangles highlight CDKs and CDKLs of *Arachis duranensis*

To study the phylogenetic relationship of *CDK* and *CDKL* gene family, we constructed an unrooted phylogenetic tree using the 133 CDKs and CDKL proteins across the three *Arachis species* and model plant Arabidopsis. Apart from conserved cyclin binding motifs, CDKs and CDKLs were divided into eight main groups in Arabidopsis based on the similarity of protein sequences (Fig. 1). Here, in general, members of most of the CDK groups were found in all three *Arachis species*. CDKA group contained four CDKs of *A. hypogaea*, two from *A. duranensis* and one from *A. ipaensis*. CDKB group contained four CDKs (two each of CDKB1 and CDKB2) of *A. hypogaea*, two each from both *A. duranensis* and *A. ipaensis*. CDKC group contained two CDKs of *A. hypogaea* and one each from both *A. duranensis* and *A. ipaensis*. CDKD group contained four CDKs of *A. hypogaea*, two from both *A. duranensis* and *A. ipaensis*. CDKE group contained two CDKs of *A. hypogaea* and one each from both *A. duranensis* and *A. ipaensis*. CDKF group contained two CDKs of *A. hypogaea*, one from *A. duranensis* and two from *A. ipaensis*. CDKG group contained six CDKs of *A. hypogaea* and three each from both *A. duranensis* and *A. ipaensis* (Fig. 1). The remaining clade was CDKL family, containing twenty-eight members of *A. hypogaea* and fourteen each from both *A. duranensis* and *A. ipaensis*. In conclusion, the phylogenetic analysis revealed that CDKs and CDKLs of peanuts showed similar phylogenetic relationship with Arabidopsis.

### Gene structure and motif composition of *CDK* and *CDKL* gene family in wild-type and cultivated peanuts

**Fig. 2:**
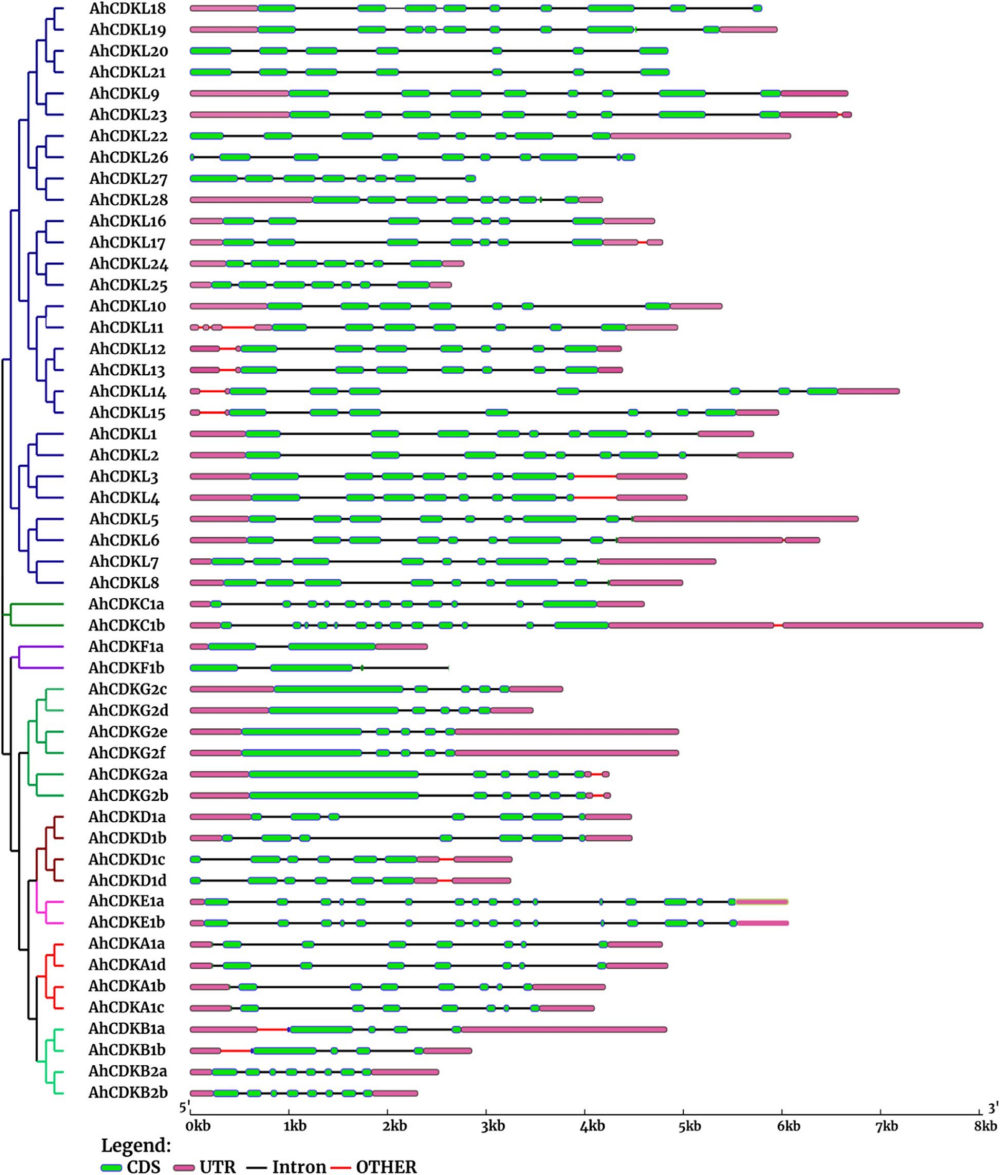
Phylogenetic tree and gene structure analysis of 52 CDKs and CDKLs of *Arachis hypogaea*. The phylogenetic tree was constructed using full-length amino acid sequences of CDKs and CDKLs proteins of *Arachis hypogaea* using MEGA 7.0 with 1000 bootstrap replicates. Different colored lines indicate diverse groups. The exon–intron architecture highlights exons as green boxes and introns as black lines and magenta color box represents both 5' and 3' UTR regions

Gene structural analysis indicated that the coding regions of all *CDK* and *CDKL* were interrupted by 1–15 introns in *A. hypogaea*, whereas in *A. duranensis* and *A. ipaensis*, intron number varied between 1–12 and 1–10, respectively. The variation of exon–intron structures in *CDK* and *CDKL* may be connected to their diverse physiological functions. Among the eight CDK classes, gene structure of *CDKAs* was more conserved compared to other groups, and all contained 7 introns in *A. hypogaea*. In addition, one *CDKF* gene had two exons and two *CDKE* genes possessed sixteen exons, indicating group of genes with the least and most exons, respectively in *A. hypogaea*. According to Fig. 2 in this species fourteen members were found to have 7 exons, while two members had 10 exons, nine had 9 exons, eleven had 8 exons, four had 6 exons, four had 5 exons and three had 4 exons. Two *CDKC* genes had 13 and 12 exons respectively, and only one *CDK* gene (*CDKF1a*) was interrupted by only one intron. In *A. duranensis*, eight members were found to have 8 exons, while six members had 7 exons, five had 9 exons and two had 5 exons (Fig. S5A). In *A. ipaensis*, eight members were found to have 8 exons, while seven members had 7 exons, three had 9 exons, two had 5 exons and two had 2 exons (Fig. S5B). Although, an enormous variation of exon and intron sizes were observed between *CDK* and *CDKLs*, genes within the same group mostly exhibited similar gene structures with regard to their distribution patterns, number and length of introns/exons in all three genomes, which corroborated with the results of our phylogenetic analysis. Besides, alteration of exon–intron structures in *CDK* and *CDKL* genes may be related to their physiological functions.

**Fig. 3:**
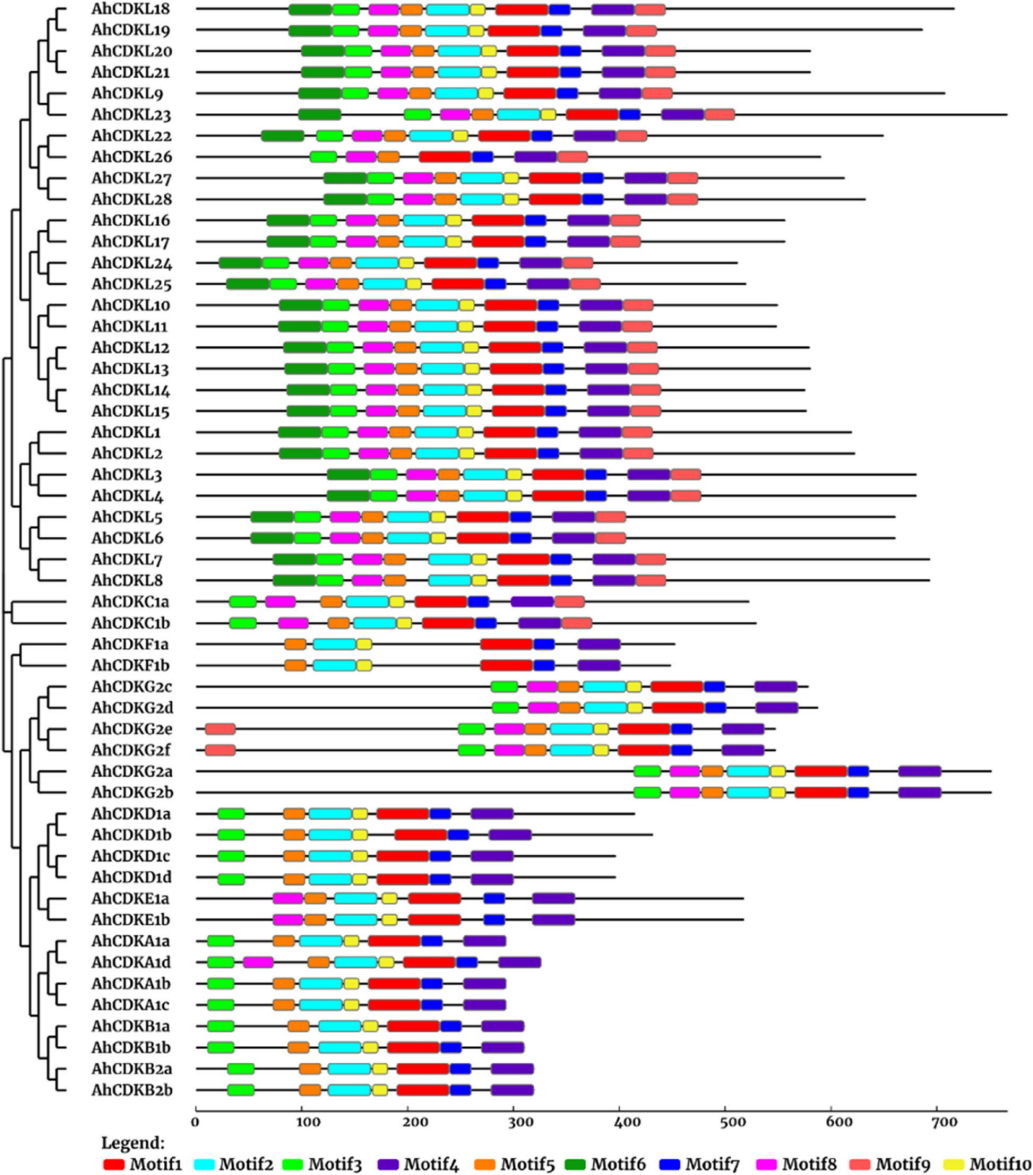
Phylogenetic tree and protein motif structure analysis of 52 CDKs and CDKLs of *Arachis hypogaea*. The phylogenetic tree was constructed using MEGA 7.0 with 1000 bootstrap replicates. The identified protein motifs are represented by colored boxes (Motif 1-Motif 10). Black lines indicate relative protein lengths. Sequence information for each motif is provided in supplementary files

To further dissect the functions of CDK and CDKL proteins, their protein domains were analysed. The MEME program (https://meme-suite.org/meme/tools/meme) discovered ten conserved motifs among all proteins of three genomes and the schematic distribution of these motifs was plotted (Fig. 3). Among them, motif 1 was uniformly found across all the CDK and CDKL proteins. Particularly, all 52 proteins in *A. hypogaea* possessed motifs 1, 2, 4, 5, 7, 10; among them ten discovered motifs were present in all CDKL proteins except AhCDKL26 (Motif 2, 6 and 10 absent), suggesting a functional similarity among CDKL proteins (Fig. S6). The motif 6 was absent in all CDKs, whereas motif 9 was limited to CDKCs (AhCDKC1a and AhCDKC1b) and CDKGs (AhCDKG2e and AhCDKG2f). All CDKs except CDKEs and CDKFs possessed motif 3, whereas CDKs other than three CDKAs, CDKBs, CDKDs and CDKFs had motif 8. A comparable motif distribution pattern was detected among CDKs and CDKLs of *A. duranensis* and *A.ipaensis*, suggesting motif arrangement corroborated with phylogenetic analysis (Fig. S7, Fig. S8, Fig. S9A, Fig. S9B). Alteration of the distribution of motifs among all CDK and CDKL proteins indicated their functional divergence.

### Chromosomal location, duplication events and synteny analysis of *CDK* and *CDKL* gene family in wild-type and cultivated peanuts

**Fig. 4:**
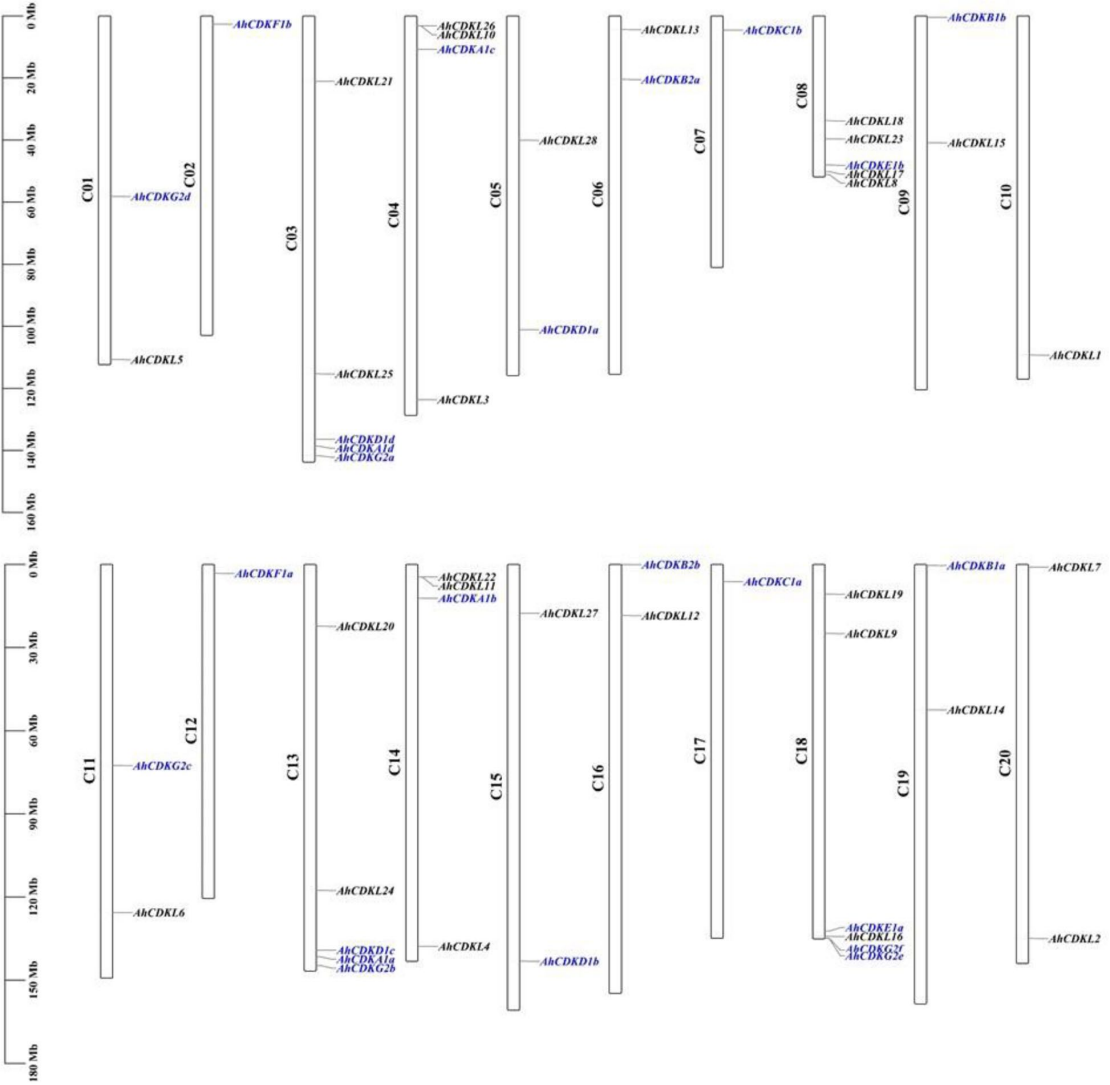
The distributions of 52 *CDK* and *CDKL* genes of *Arachis hypogaea* across 20 chromosomes. The *CDKA-CDKG* group genes are depicted in blue, whereas the *CDKL* genes are highlighted in black. The values represent the relative start positions (Mbp) of each gene

Genomic distribution of *CDK* and *CDKL* genes showed variation with respect to their chromosomal locations. Out of 52, 49 *CDK* and *CDKL* genes were mostly distributed among the 20 chromosomes in cultivated peanuts, while the remaining 3 genes were localized to unmapped scaffolds (Fig. 4) in the genome. The distribution of the total fifty-two *CDK* and *CDKL* genes varied among the 20 chromosomes in cultivated peanuts. Chromosome 18 harbored the maximal number of genes that is six, whereas, chromosomes 2, 7, 10, 12 and 17 had a single gene. Chromosomes 3, 8 and 13 had five genes, followed by chromosomes 14 and 4 with four, and chromosomes 1, 5, 6, 9, 11, 15, 16, 19 and 20 with two genes. The parental species *A. duranensis* and *A. ipaensis* comprised of 10 chromosomes each with uneven distributions of the putative *CDK* and *CDKL* genes (Fig. S10, Fig. S11) and at least one predicted gene was present on each chromosome of the two species. Chromosome 3 of *A. duranensis* had the highest number of genes including four AdCDKs and two AdCDKLs, followed by chromosome 8 (five genes), chromosome 4 (four genes), chromosome 1 (three genes) and chromosomes 5 and 6 (two genes), respectively. But only one candidate gene remained in chromosomes 2, 7, 9 and 10. In *A. ipaensis*, the maximum number of genes being detected on chromosome 8 (five genes), followed by chromosomes 3 and 4 (four genes), chromosomes 1, 2, 5, 6, 9 and 10 (two genes) and chromosome 7 (one gene), respectively. In particular, longer chromosomes do not necessarily contain more *CDK* and *CDKL* genes, demonstrating that the number of *CDK* and *CDKL* genes on each chromosome is unrelated to length. In *A. hypogaea*, Pearson’s r and p-value are 0.039 and 0.868 respectively, whereas *A. duranensis* Pearson’s r and p-value are 0.037 and 0.919 respectively, and *A. ipaensis* Pearson’s r and *p*-value are -0.027and 0.940 respectively. None of the above correlations is significant.

**Fig. 5:**
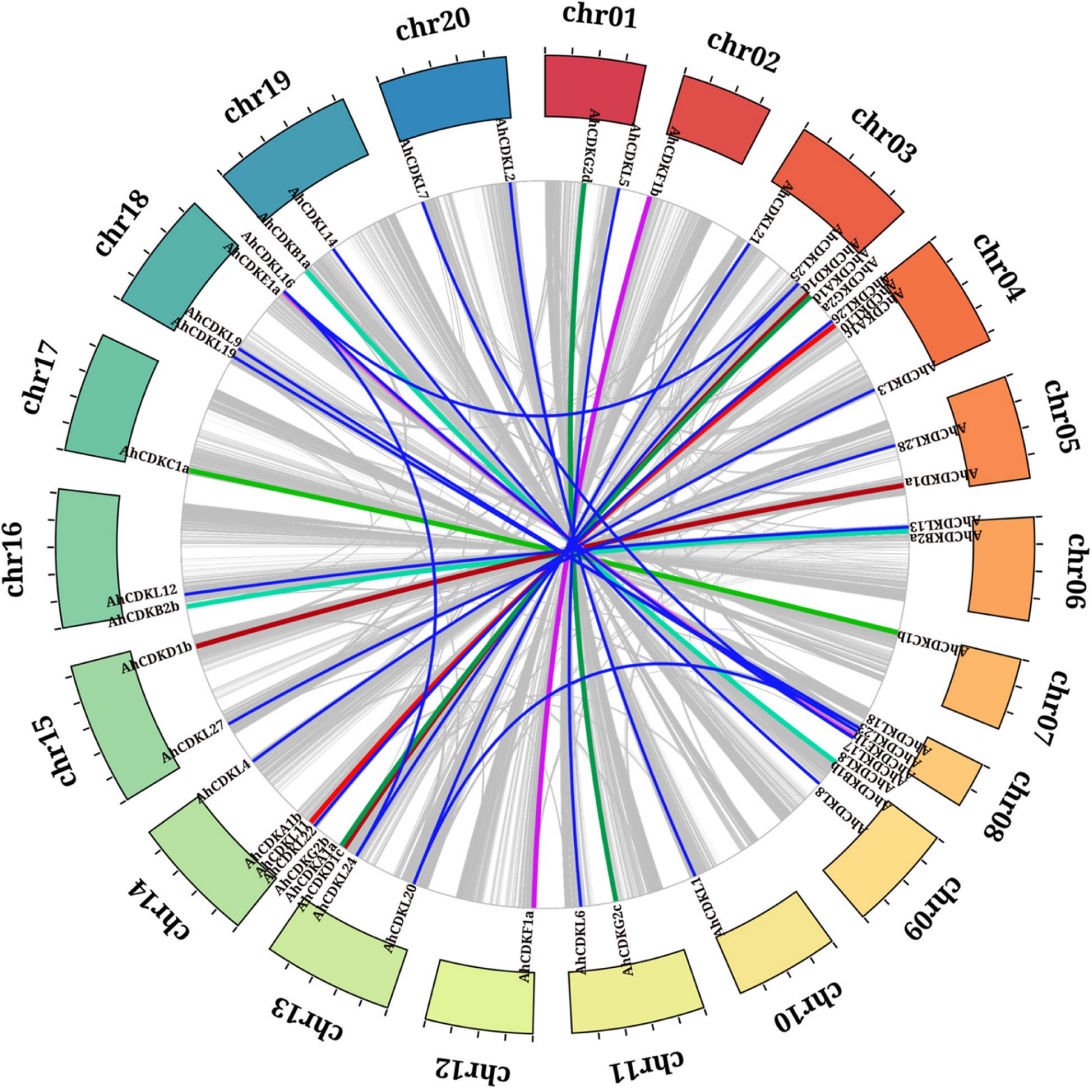
Analysis of duplication events of *CDK* and *CDKL* genes of *Arachis hypogaea*. The colored blocks represent the 20 chromosomes of *A. hypogaea* and the block size corresponds to the relative chromosome lengths. Segmental duplication gene pairs are linked by lines. The *CDK* and *CDKL* gene pairs are differently colored based on the respective CDK groups, and the non-CDK duplication pairs are shown as grey lines

Gene duplication plays an important role in the expansion of gene families by including genetic novelty and subsequent evolution. Nearly fifty percent (47.6%) of the total genes in the peanut genome were segmentally duplicated. Other types of duplications like dispersed, proximal and tandem constituted 21.7%, 4.5% and 3.4% of the total genes, respectively. Additional 22.7% are singletons. In order to figure out the origins of the *CDK* and *CDKL* genes in cultivated peanuts, we performed syntenic analysis with MCScanX. The genome duplication study identified 27 *CDK* and *CDKL* segmental gene duplication pairs (alignment blocks with E-value < 1e-100). Tandem duplications were absent among the peanut CDK genes and only a single instance of proximal duplication (*AhCDKG2e*) was identified. A total of 27 gene pairs distributed among the 20 chromosomes with 50 *AhCDK* and *AhCDKL* genes were categorized as segmental duplications, indicating its significance in the evolution of allotetraploid peanut *CDK* and *CDKL* genes (Fig. 5). Altogether it suggested that segmental duplication has played a major role in *CDK* and *CDKL* genes evolution in *A. hypogaea*.

**Fig. 6:**
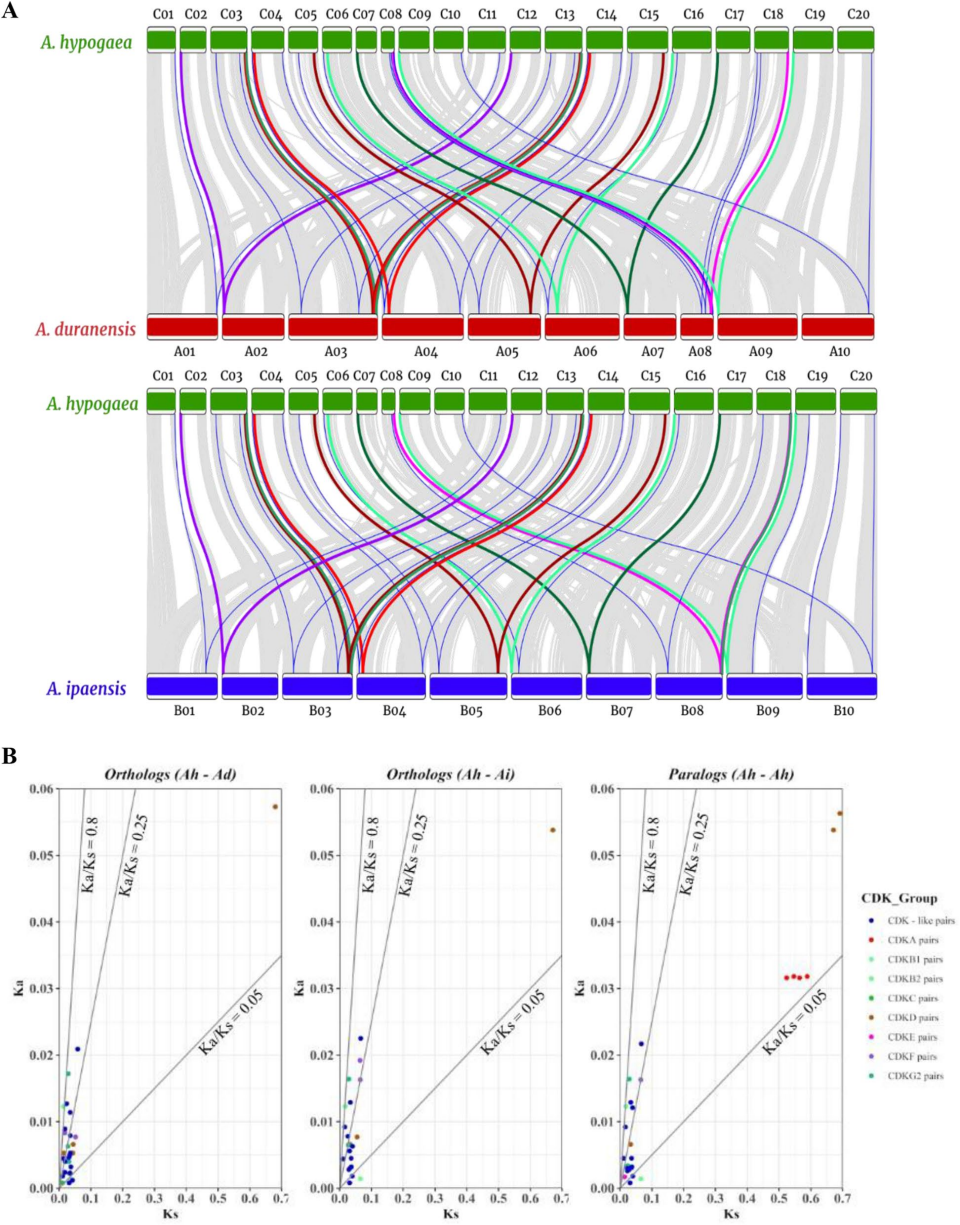
Synteny analysis and Ka/Ks ratios of *CDK* and *CDKL* genes between cultivated peanut and its diploid parents. **A** The collinear gene pairs between *A. hypogaea* ­ *A. duranensis* and *A. hypogaea—A. ipaensis* were identified by MCscanX. The grey shades in the background indicate the collinear blocks between *A. hypogaea* ­ *A. duranensis* and *A. hypogaea—A. ipaensis*. Other different colored lines correspond to the various CDK group gene pairs. **B** The Ka/Ks ratios of the predicted *CDK* and *CDKL* genes orthologs and paralogs. The Ka/Ks ratios of 0.05, 0.25 and 0.8 are depicted as black lines. The different dot colors represent gene pairs belonging to diverse CDK groups

The MCScanX results also contributed to detecting syntenic and collinear relationships of *A. hypogaea* with the diploid parents. We constructed collinearity syntenic maps of *A. hypogaea* along with the two-parent species, *A. duranensis* and *A. ipaensis*, respectively. The *CDK* gene pairs within the significant collinear blocks (zero e-value) were identified in *A. hypogaea* – *A. duranensis* (44 pairs) and *A. hypogaea* – *A. ipaensis* (41 pairs) maps. We found that the collinearity blocks containing *CDK* genes were mainly distributed on the chromosomes, C03 and C13 of *A. hypogaea* (Fig. 6A). Thirty-seven *CDK* genes of *A. hypogaea* were paired in both *A. duranensis* and *A. ipaensis* collinear blocks, suggesting a closer evolutionary connection among the three genomes.

To further explore the dynamics of evolution of *CDK* and *CDKL* genes in *A. hypogaea*, Ka (non-synonymous substitutions per site), Ks (synonymous substitutions per site) and Ka/Ks ratios for each duplication pair estimation were carried out (Table S2). The Ka/Ks ratios of all peanut *CDK* and *CDKL* pairs within peanut (paralogs) and between peanut and its progenitors (orthologs) among three different genomes were less than 1, most of which were less than 0.5, indicating that these paralogs and orthologs underwent strong purifying selection after gene duplication events (Fig. 6B).

### Analysis of cis-acting elements in the promoter regions of *CDK* and *CDKL* gene family in wild-type and cultivated peanuts

**Fig. 7:**
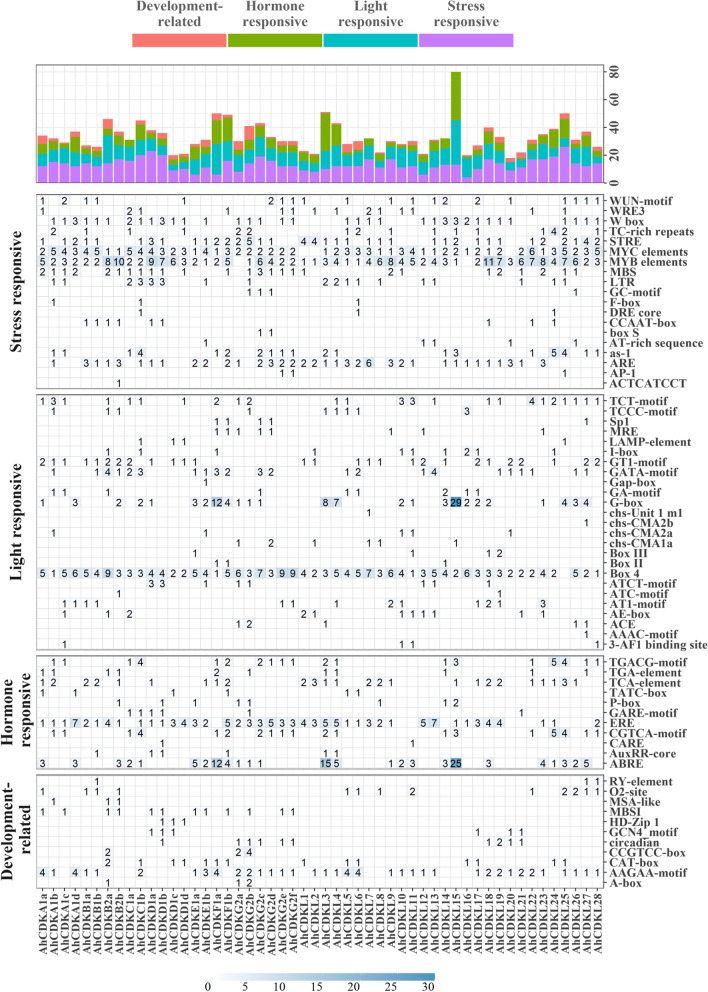
The *CDK* and *CDKL* genes promoter cis-elements distribution in *Arachis hypogaea*. In upper panel, different colors was used to recognize various types of promoter cis-elements, like development related, phytohormone-responsive, light responsive and stress responsive. In lower panel, number of each cis-acting element of the *CDK* and *CDKL* gene promoter region (1.5 kb upstream of the translation start site)

To elucidate the regulatory mechanisms governing *CDK* and *CDKL* genes in response to growth and development, stress and hormone signaling, a 1500-bp 5′-upstream noncoding region sequence of each *CDK* and *CDKL* genes from three genomes was isolated to predict cis-elements. In addition to basic cis-elements, such as the promoter-related elements (CAAT-box and TATA-box), multiple categories of regulatory elements were identified in all promoters, including hormone and light-responsive elements, development and stress-related elements, site binding related, and other elements (Fig. 7, Fig. S12, Fig. S13). The analysis further focused on the development-related, stress-responsive and phytohormone-responsive elements as they are key players in transcriptional regulation. The development-related cis-elements identified in promoters of the putative genes across the three species included zein metabolism regulation (O2-site), cell cycle regulation (MSA-like), seed-specific regulation (RY elements), meristem expression (CAT-box), meristem specific activation (CCGTCC box), endosperm expression (GCN4 motif), circadian and palisade mesophyll cell differentiation (HD-Zip 1) elements (Fig. S14). The GCN4 motif, O2-site and CAT-box elements remained in 6, 13 and 14 putative gene promoters, respectively, in *A. hypogaea*, whereas these numbers were 3, 6 and 7 in *A. duranensis* and 3, 8 and 6 in *A. ipaensis*. Predominantly, HD-Zip 1 elements were present at the upstream of a few *CDKD* genes, like *AhCDKD1b*, *AhCDKD1c*, *AhCDKD1d*, *AdCDKD1a*, *AiCDKD1a* and *AiCDKD1b*, whereas MSA-like elements were identified at the promoter regions of *CDKA* (*AhCDKA1b* and *AiCDKA1*), *CDKB2* (*AhCDKB2a*, *AhCDKB2b*, *AdCDKB2* and *AiCDKB2*) and *CDKG* (*AdCDKG2c*) genes.

The stress-responsive elements like dehydration responsive elements (DRE core), low temperature responsive (LTR), anaerobic responsive elements (ARE), abiotic and biotic stress responsive (F-box), drought inducible MYB binding site (MBS), MYB, MYC, STRE, W-box, ACTCATCCT, AP1, WUN-motif, TC-rich repeats etc. were identified in the putative gene promoter regions of *CDK* and *CDKL* genes of the three genomes (Fig. 7, Fig. S12, Fig. S13, Fig. S14) The number of LTR element containing gene promoters were 18, 10 and 11 in *A. hypogaea*, *A. duranensis* and *A. ipaensis*, respectively, indicating that these *CDKs* and *CDKLs* participated in cold stress response. Although the majority of the gene promoters had MYB, MYC and STRE elements, F-box elements exclusively remained upstream of *CDKA* (*AhCDKA1b* and *AiCDKA1*), *CDKC* (*AhCDKC1b* and *AdCDKC1*) and *CDKL* (*AhCDKL6* and *AiCDKL3*) genes. In addition, most of the *CDKA*, *CDKB2*, *CDKD*, *CDKG2* and *CDKL* groups contained MBS elements. In contrast, the DRE core element predominantly remained in the promoter regions of *CDKC* (*AhCDKC1b* and *AdCDKC1*), revealing that *CDKs* may be related to the tolerance of abiotic stresses, especially drought stress in plants.

Hormone-responsive elements including auxin-responsive (AuxRR-core and TGA-element), ABA-responsive (ABRE), SA-responsive (TCA element), gibberellin-responsive (P-box and GARE motif) and MeJA-responsive (TGACG-motif and CGTCA-motif) elements were found in *CDK* and *CDKL* gene promoters (Fig. 7, Fig. S12, Fig. S13, Fig. S14). In *A. hypogaea*, *CDK* and *CDKL* genes with AuxRR-core, TGA-element, TCA-element, P-box, GARE motif and TGACG motif were 6, 11, 25, 8, 6 and 19, respectively. In *A. duranensis*, the *CDK* and *CDKL* genes with AuxRR-core, TGA-element, TCA-element, P-box, GARE motif and TGACG motif were 4, 6, 15, 3, 3 and 9, respectively, whereas in *A. ipaensis*, these numbers were 2, 9, 13, 5, 3 and 10. Overall, these diverse response elements in *CDK* and *CDKL* genes indicated their potential integral roles in different biological processes.

### Expression profile and correlation analysis of *CDK* and *CDKL* gene family in different tissues in cultivated peanuts

**Fig. 8:**
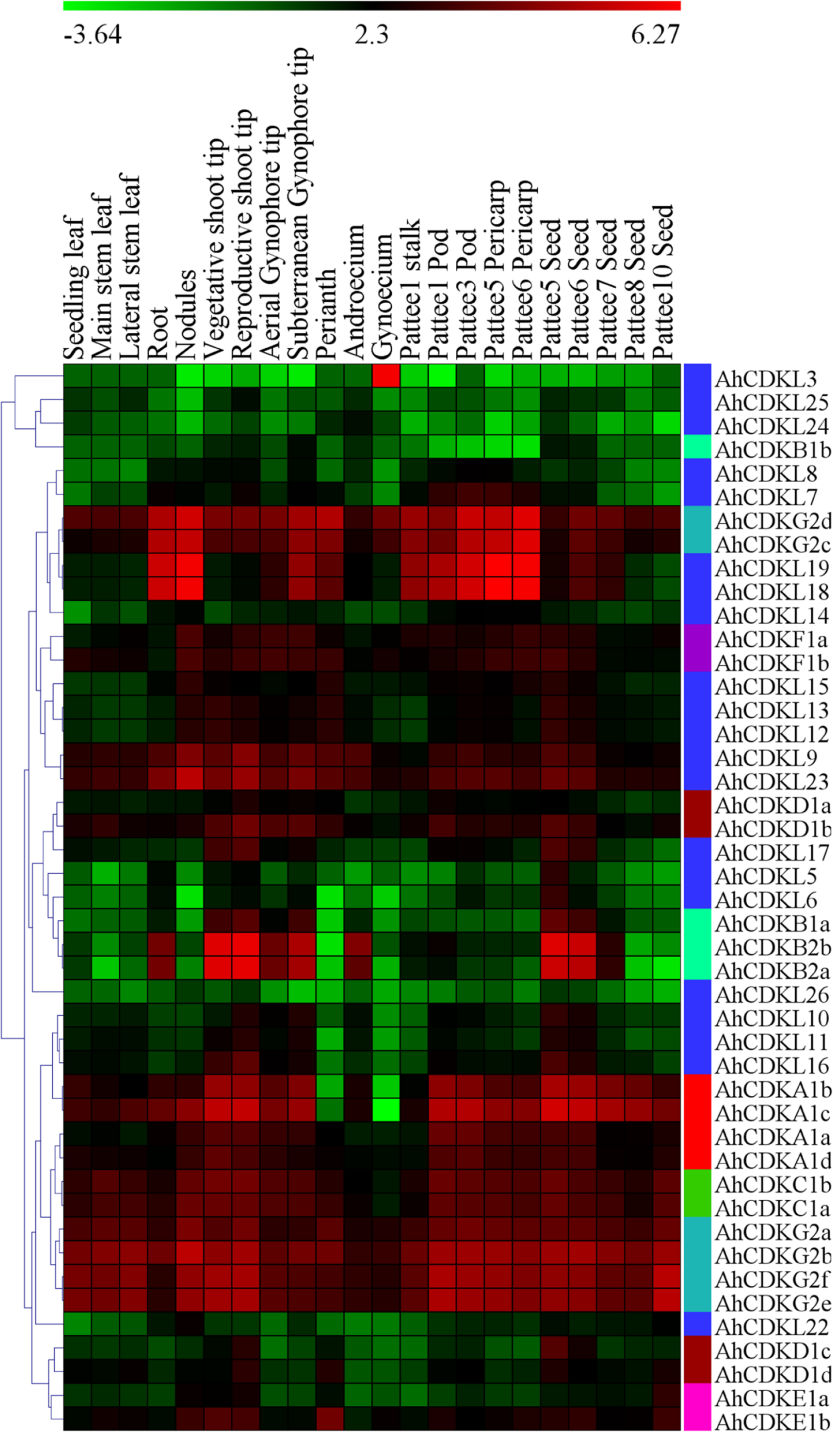
Expression profiles of *CDK* and *CDKL* genes of *Arachis hypogaea* *TIFY* genes in different vegetative and reproductive tissues. Transcript profiles of 45 putative peanut *CDK* and *CDKL* genes in 22 different tissues at various development stages. The FPKM values obtained were normalized by log_2_ transformation and the heatmap constructed using the MeV software. The block colors represent the expression levels, like green, black and red correspond to low, medium and high expression values

Spatiotemporal expression of transcript is primarily linked with the biological function of gene. The expression patterns of available 45 *CDK* and *CDKL* genes were investigated using a standard transcriptome analysis procedure based on public transcriptomic data of different tissues of *A. hypogaea*, including leaves of lateral stem, main stem and seedling, vegetative and reproductive shoot tip, root, nodule, perianth, stamen, pistil, peg tip aerial, peg tip below soil, fruit pattee, pericarp pattee and seed pattees [40, 41]. Among the 45 genes, about 19 genes in vegetative shoot tip, 21 genes in reproductive shoot tip, 10 genes in the roots and 15 genes in the nodules exhibited the higher transcript levels (FPKM > 10). All *CDKG* and *CDKC* genes along with two *CDKA* (*AhCDKA1a* and *AhCDKA1d*) and two *CDKL* (*AhCDKL9* and *AhCDKL23*) were constitutively expressed (FPKM > 1) in all the 22 tissues suggesting their substantial role in peanut growth and development. A few of the genes exhibited tissue-specific expressions, for example, *AhCDKL3* had a high expression in gynoecium, whereas *CDKB2* genes showed significant expression in shoot tips (vegetative and reproductive) and during the early seed stages (Pattee 5 and Pattee 6) (Fig. 8). In addition, a number of genes showed a high expression in specific tissues, for instance *AhCDKG2b* in the main stem leaf, *AhCDKG2b* in the lateral stem leaf, *AhCDKL18* and *AhCDKL19* in the root, *AhCDKL18* and *AhCDKL19* genes in nodule, *AhCDKG2d* genes in perianth and *AhCDKB2b* genes in androecium. The transcript levels of a few *CDK* and *CDKL* genes significantly decreased in the lateral stem leaf, main stem leaf, perianth, stamen and pistil compared to other tissues. Although *AhCDKA1b* and *AhCDKA1c* genes exhibited high expression in most tissues, a notable reduction of their expression was observed in perianth and gynoecium (Fig. 8).

**Fig. 9:**
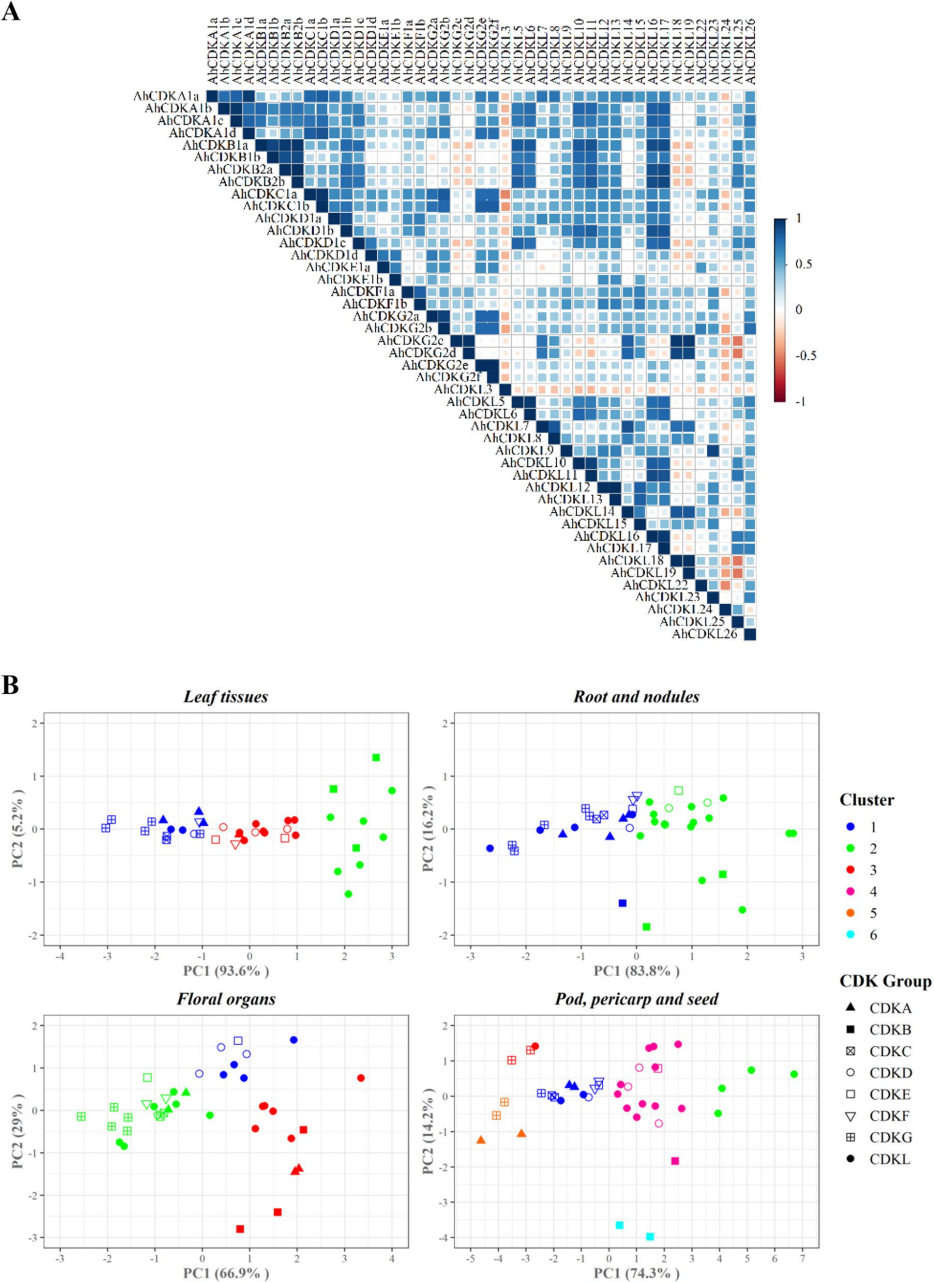
Correlation and clustering analysis of the *CDK* and *CDKL* genes of *Arachis hypogaea*. **A** The expression values in different tissues was used to determine Pearson’s pairwise gene correlations of the *CDK* and *CDKL* genes. Blue and red colors represent positive and negative correlations with significant p-values (*p*-value < 0.05), respectively. The color intensities are proportional to the correlation coefficients (r) **B** PCA based k-means cluster plots classified CDK group of genes into expression-based clusters in leaf tissues, root and nodules, floral organs. and pod, pericarp and seed. The optimal number of clusters in each case were determined using the NbClust package

Pairwise gene correlations analysis of *CDK* and *CDKL* genes using expression values in 22 different tissues with *p*-value < 0.05 was depicted (Fig. 9A). Most genes in the similar *CDK* group showed very strong positive correlations (Pearson’s r > 0.85, *p* < 0.05) like *AhCDKA1b/AhCDKA1c*, *AhCDKB1a/AhCDKB1b*, *AhCDKB2a/AhCDKB2b*, *AhCDKC1a/AhCDKC1b*, *AhCDKD1a/AhCDKD1b*, *AhCDKG2c/AhCDKG2d* and *AhCDKL5/AhCDKL6* (Fig. 9A). Besides, certain unrelated *CDK* gene pairs also showed significant positive correlations like *AhCDKA1d/AhCDKC1b*, *AhCDKB1a/AhCDKL11*, *AhCDKB2b/AhCDKL16*, *AhCDKD1b/AhCDKL17* and *AhCDKG2c/AhCDKL18*. Figure 9B represented the *CDK* gene clustering analysis (PCA based k-means clustering) using gene expressions in leaf tissues, root-nodules, floral organs and pod-pericarp-seed. Three optimal clusters were proposed based on the expression in leaf tissues, and most of the genes belonged to cluster 3 (40.5%). Clusters 1 and 2 comprised 35.7% and 23.8% genes, respectively. Genes with relatively higher expressions remained in cluster 1 (all *CDKGs* and *CDKCs*, three *CDKA*, *AhCDKD1b*, *AhCDKF1b* and two *CDKLs*), whereas cluster 2 genes (all *CDKBs* and seven *CDKLs*) showed lower expressions. In contrast, cluster 3 genes (all *CDKEs*, most *CDKDs*, *AhCDKA1a*, and *AhCDKF1a*) displayed intermediate expressions. Root and nodule gene clustering identified two optimal clusters with both clusters having a relatively equal number of genes (51% and 49%, respectively), where cluster 1 comprised the majority of the *CDK* genes and cluster 2 had most of the *CDKL* genes. Three optimal clusters were proposed considering the gene expressions in floral organs. Cluster 2 comprised 54% of genes with higher expression values, whereas cluster 3 and cluster 1 had 27% and 19% of the genes, respectively. The pod-pericarp-seed expressions identified six *CDK* gene with clusters 1–6 encompassing 25.6%, 9.3%, 9.3%, 40%, 11.6%, and 4.6% genes, respectively. Cluster 1 genes (*AhCDKA1a*, *AhCDKA1d*, *AhCDKC1a*, *AhCDKC1b*, *AhCDKD1b*, *AhCDKE1b*, *AhCDKF1a*, *AhCDKF1b*, *AhCDKG2a*, *AhCDKL9*, and *AhCDKL23*) had relatively higher expression values compared to other gene clusters. Cluster 2 and 6 had only *CDKL* and *CDKB2* genes, respectively. Overall results indicated that these genes might play a role in many aspects of peanut vegetative and reproductive organ development (Fig. 9B).

### Response of *CDK* and *CDKL* genes under abiotic stresses

**Fig. 10:**
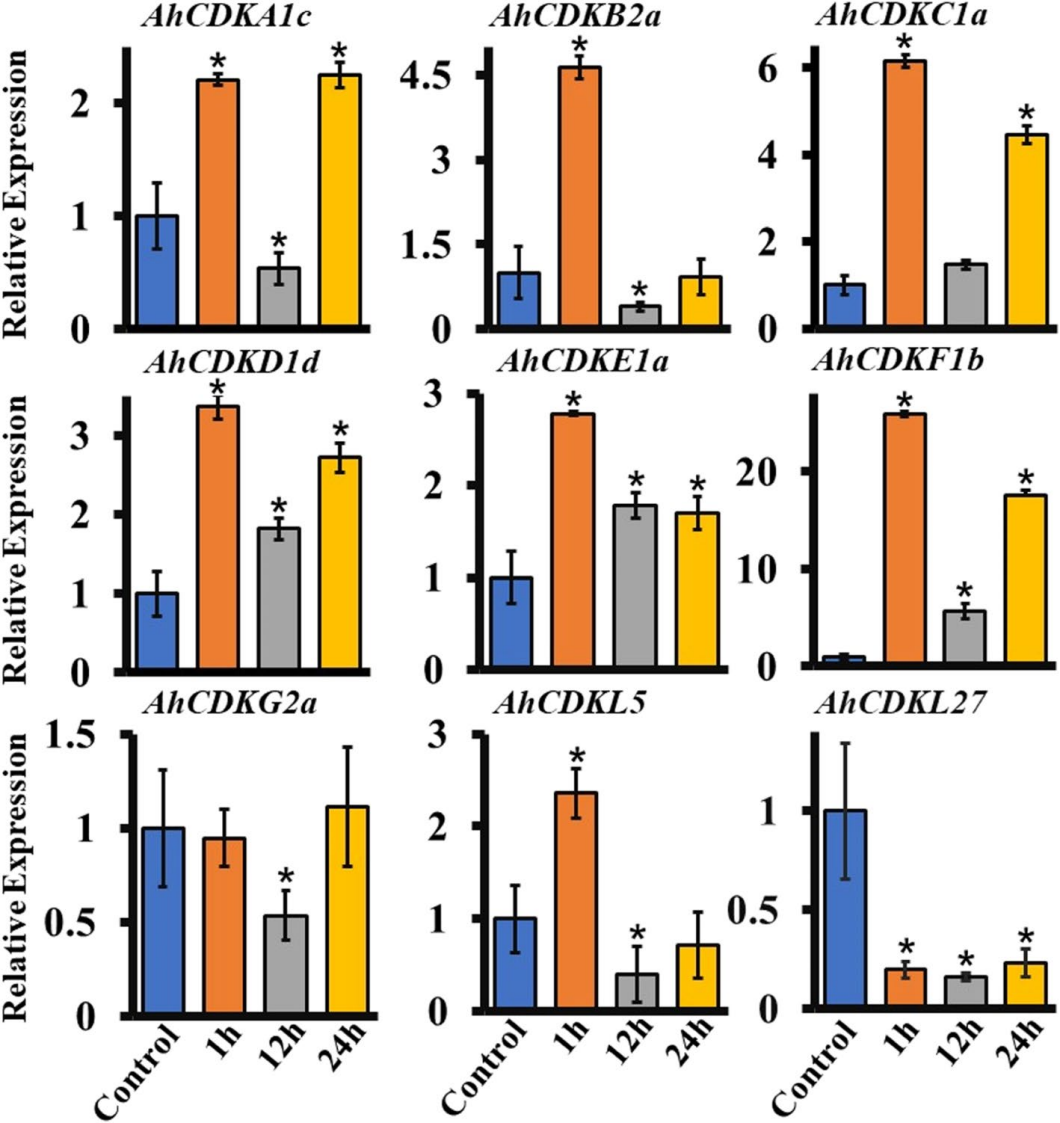
Expression patterns of different *CDK* and *CDKL* genes in roots of peanut under ABA treatment. Total RNA was extracted from roots at different time points after 100 μM ABA treatment (1 h, 12 h, 24 h) and without ABA treatment (Control or 0 h) and subjected to quantitative RT-PCR analysis. *Ubiquitin* of peanut as an internal reference gene, the relative expression level was calculated by 2^−ΔΔCt^. The data are the mean ± SE of three independent biological samples. *, *p* < 0.05

**Fig. 11:**
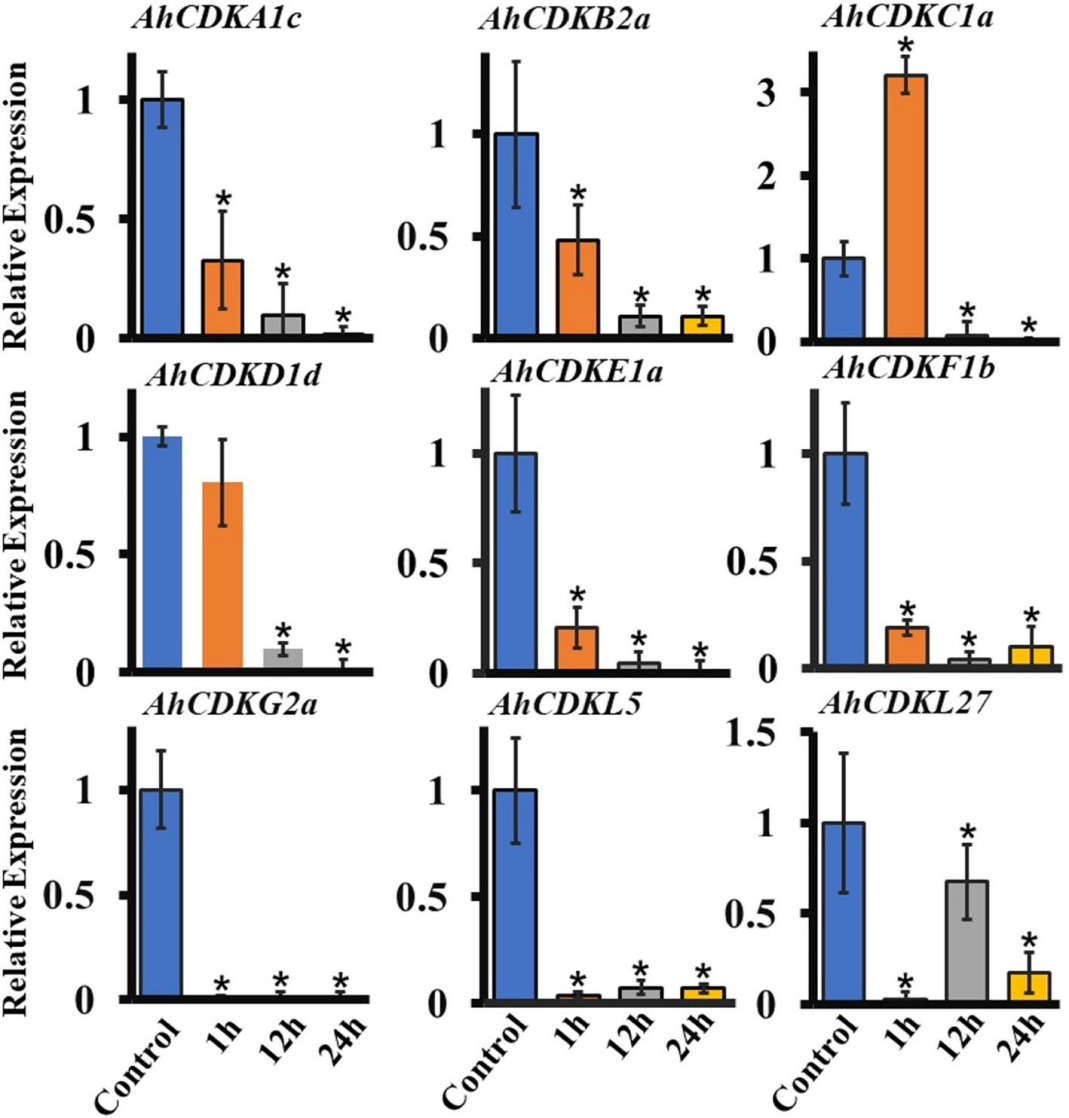
Expression patterns of different *CDK* and *CDKL* genes in roots of peanut under PEG treatment. Total RNA was extracted from roots at different time points after PEG (20%) treatment (1 h, 12 h, 24 h) and without PEG treatment (Control or 0 h) and subjected to quantitative RT–PCR analysis. *Ubiquitin* of peanut as an internal reference gene, the relative expression level was calculated by 2^−ΔΔCt^. The data are the mean ± SE of three independent biological samples. *, *p* < 0.05

**Fig. 12:**
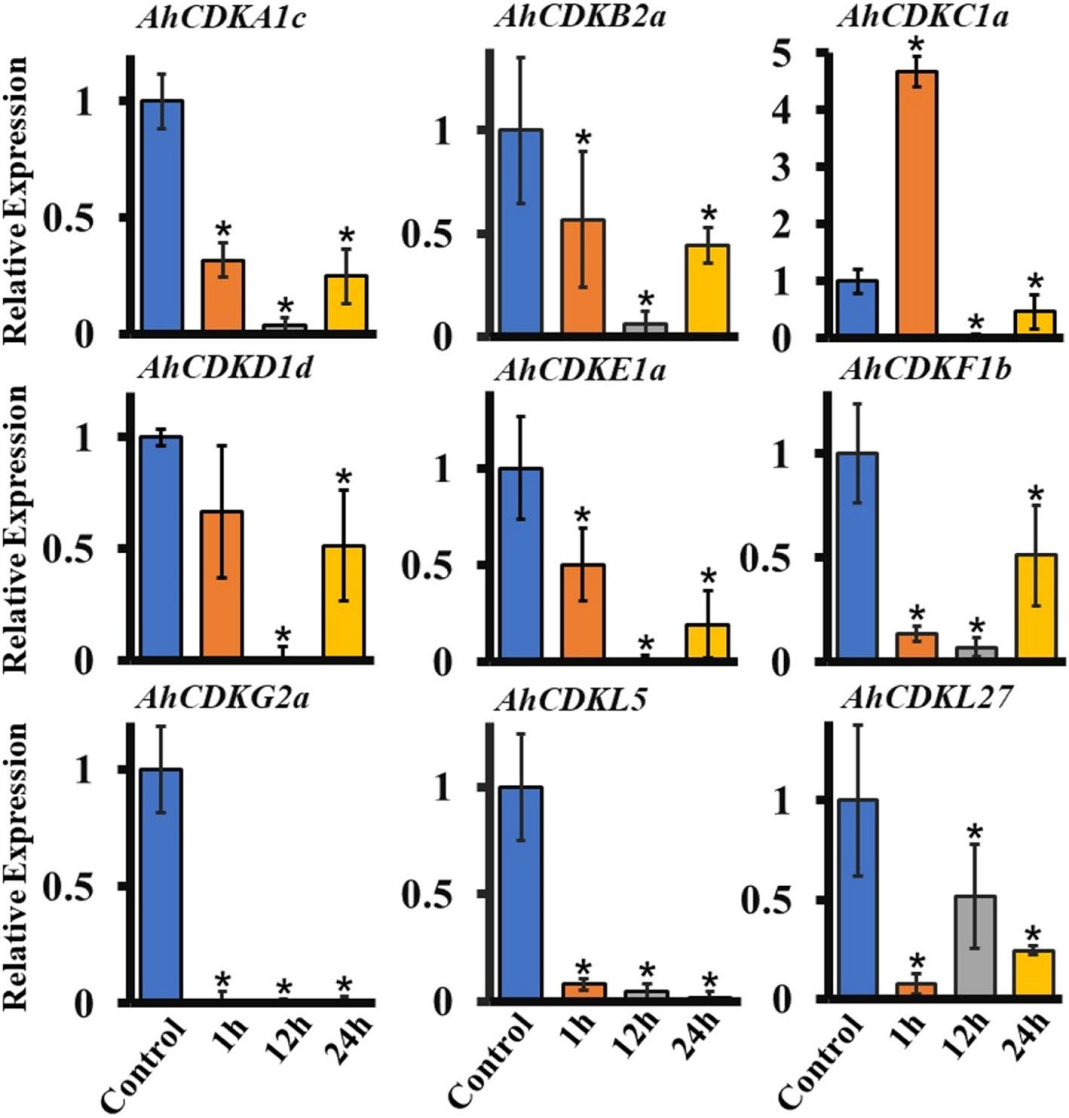
Expression patterns of different *CDK* and *CDKL* genes in roots of peanut under mannitol treatment. Total RNA was extracted from roots at different time points after mannitol (200 mM) treatment (1 h, 12 h, 24 h) and without mannitol treatment (Control or 0 h) and subjected to quantitative RT-PCR analysis. *Ubiquitin* of peanut as an internal reference gene, the relative expression level was calculated by 2^−ΔΔCt^. The data are the mean ± SE of three independent biological samples. *, *p* < 0.05

Global climate change like prolonged drought seriously impacts the growth and yield of peanuts worldwide [42]. Among the several abiotic stresses, drought predominantly affects cell cycle progression in plants by reducing the mitotic activity of cells thus limiting plant growth and crop yield [43, 44]. To explore expression patterns of the *CDK* and *CDKL* genes in response to drought treatments, we analysed expression pattern of nine selected genes representing different groups of *CDK* and *CDKL* at different time points (1 h, 12 h, and 24 h) after ABA, PEG and mannitol treatment. *AhCDKA1c, AhCDKB2a, AhCDKC1a, AhCDKD1d, AhCDKE1a, AhCDKF1b* and *AhCDKL5* genes were upregulated following 1 h after ABA treatment, suggesting that these genes might play a positive role in response to ABA (Fig. 10). In contrast, *AhCDK27* gene was significantly downregulated in response to ABA, and even AhCDKG2a gene was not modulated by ABA. The expression levels of most of these *CDK* and *CDKL* genes decreased gradually after prolonged exposure to ABA, indicating a complex regulatory expression network. These *CDK* and *CDKL* genes responded to PEG and mannitol treatments to a greater extent than ABA treatment. All the genes were drastically downregulated in response to PEG and mannitol at both early and late time points, suggesting that *CDK* and *CDKL* genes play negative roles under PEG and mannitol treatments (Fig. 11, Fig. 12). Only *AhCDKC1a* gene expression was induced after 1 h PEG and mannitol treatments, but its expression was severely reduced after 12 or 24 h of PEG and mannitol treatments.

## Discussion

Cell proliferation is a tightly controlled process during which two identical daughter cells are generated after DNA replication. A coordinated entry into the cell cycle monitors cell proliferation, and transitions through the cell cycle is mostly encouraged by cyclin-dependent kinases (CDKs)[3, 45, 46]. Typically, *CDK* and *CDKL* genes activate cell multiplication after binding with multiple cyclins, but *CDK* and *CDKL* genes that are activated by single cyclin can modulate transcriptional regulation [47]. In eukaryotes, CDKs have undergone a remarkable degree of evolutionary divergence and specialization to play their functional role in a complex way via phosphorylation and/or dephosphorylation in response to several developmental and environmental cues [48]. The *CDK* and *CDKL* genes lead cell cycle progression throughout plant development by modulating mitotic and meiotic cell division, endoduplication, cytokinesis, microtubule dynamics and alternative splicing circuit [24, 29, 49–53]. The members of *CDK* and *CDKL* genes have so far been identified in the model plant Arabidopsis, as well as other plants, such as rice, *Gossypium species*, *Phalaenopsis aphrodite*, maize, soybean, and Antirrhinum [54–60].

A comprehensive genome-wide identification of plant *CDK* and *CDKL* family genes from several crop plants is crucial before their functional characterization. Genome-wide these CDK gene family have only been identified from Arabidopsis, rice, *Gossypium species* and *Phalaenopsis aphrodite* [54–57]. Despite the fact that peanut is an economically important oil-yielding crop in the Fabaceae family, characterization of it’s cell cycle regulatory genes has received much less attention. Therefore, their exact functions remain obscure. But recent availability of genome sequences of peanut species has facilitated the comprehensive identification of several gene families. In the current study, our detailed genome-wide analysis of *CDK* and *CDKL* family identified 52 genes from *A. hypogaea* and 26 each from *A. duranensis* and *A. ipaensis*. Based on the conserved structural domains of the *CDK* and *CDKL* genes, we classified them into eight subgroups (Fig. 1). Gene structure and conserved motif were also confirmed by phylogenetic analysis (Fig. 2, Fig. 3). Similar to Arabidopsis and *Arachis species*, eight different groups of CDKs, altogether 25 *CDK*s, were detected in rice, including 2 *CDKA*, 2 *CDKB*, 3 *CDKC*, 1 *CDKD*, 1 *CDKE*, 4 *CDKF1*, 2 *CDKG* and 10 *CDKL*s [55]. In cotton, a total of 31, 12, and 15 putative *CDK* genes were identified from three *Gossypium species* (*G. hirsutum*, *G. arboreum*, and *G. raimondii*) and all genes were divided into six different groups [56]. Total nine *CDKs*, including one member of *CDKA1*, *CDKB1*, *CDKB2*, *CDKC1*, *CDKD1*, *CDKE1*, *CDKF1*, *CDKG1*, *CDKG2* and 13 *CDKL*s were identified in *Phalaenopsis aphrodite* [57].

Gene structure analysis revealed that C and E groups contained genes with more than ten introns, whereas A, B2, D1 and CDKL group members were interrupted by six to nine introns. But F and B1 groups had genes with fewer introns. Presumably, C and E groups may comprise primitive genes as the rate of intron deletion was faster than that of intron gaining after segmental duplication during land plant evolution. Moreover, increase and loss of intron numbers are one of the causes of diversification [61]. In addition, increase of intron numbers may be advantageous for evolution facilitating easier adaptation of plants to the fluctuating climate conditions. In contrast, the presence of fewer introns is presumably favourable for fast response to the environment [62]. Although CDK groups of *A. hypogaea* showed 6 conserved motifs, CDKL members possessed all 10 motifs suggesting that CDKL members are structurally varied from CDKs. In addition, the occurrence of motif 1 in all proteins indicated a conserved function of CDK and CDKL proteins in *Arachis species*. Furthermore, the existence of different protein motifs is the cause of the multiple groups of CDKs, thus each CDK group contains a similar type of motif. The physical map of *CDK* and *CDKL* genes of *Arachis species* have shown their distribution among all chromosomes (Fig. 4). The number of *CDK* and *CDKL* genes on the longest chromosome is not maximum, and that on the shortest chromosome is not the minimum, indicating the absence of a positive correlation between the *CDK* and *CDKL* gene numbers and length of the chromosome. A few genes may function together as an isoform of proteins as they are positioned as clusters on the chromosomes.

Gene duplications of land plants are conducive for adaptation to constantly changing environments, and these duplicated genes often undergo sub- or neo-functionalization [63]. Resulting redundant copies either show similar functions or act in a redundant manner essential for functional conservation or divergence [64, 65]. The variation in the number of *CDK* and *CDKL* genes between three different Arachis genomes may be related to gene duplication events. We identified 49 pairs of segmentally duplicated and 2 pairs of tandemly duplicated genes (Fig. 5). In maize, sorghum and other crop plants, most of the genes were segmentally duplicated, suggesting a substantial role of artificial selection during crop domestication may be one of the causes for higher numbers of segmentally duplicated genes [66–68]. Similar exon–intron structures and conserved domains were observed in most gene pairs involved in duplication, suggesting their conserveness after genome duplication. These results further corroborate the notion that gene duplication is mainly responsible for the expansion of the *CDK* and *CDKL* genes, and segmental duplication was the main cause underlying *CDK* and *CDKL* genes expansion. Synteny and collinearity analysis also revealed that segmental type of gene duplication played a key role in the expansion and diversification of the *CDK* genes in peanuts, consistent with previous studies in other species [66–68].

It is obvious that the number of *CDK* and *CDKL* genes in allotetraploid peanut *A. hypogaea*, were significantly higher from their diploid progenitors, *A. duranensis* and *A. ipaensis*. The position of *CDK* and *CDKL* genes on C03, C04, C08, C13, and C14 in *A. hypogaea* have a nice collinear relationship with the *CDK* and *CDKL* genes remained on the homologous chromosomes of *A. duranensis* and *A. ipaensis*. This synteny map also demonstrated that duplication and rearrangement of the chromosome might significantly affect the origin of allotetraploid *A. hypogaea* from its progenitors (Fig. 6A). To explicate the divergence throughout peanut evolution, orthologous and paralogs of peanut genes between *A. hypogaea*, *A. duranensis* and *A. ipaensis* were identified. Fifty-one ortholog pairs were identified between *A. hypogaea-A. duranensis* and *A. hypogaea-A. ipaensis*, whereas thirty-two paralog pairs were identified in *A. hypogaea*. Furthermore, the Ka/Ks ratios specified that *CDK* and *CDKL* genes evolved largely under purifying selection, presumably essential for adaptation to the existing environment as their evolution was significantly slow (Fig. 6B). These results further demonstrated that *CDK* and *CDKL* genes of *A. hypogaea* derived from *A. duranensis* and *A. ipaensis* have followed negative selection and may involve a slow abolition process during evolution.

We have identified cis-elements in the promoter regions of *CDK* and *CDKL* genes to understand the gene functions and regulatory mechanisms. Numerous cis-acting elements responsible for development, hormonal regulation, light and stress response were recognized in the promoters of all *CDK* and *CDKL* genes, specifying their indispensable role in almost all aspects of plant growth and development and diverse kinds of stress tolerance (Fig. 7). Besides cell cycle progression, relative abundance of multiple hormonal (ABRE, ERE, Aux-RR core, etc.) and stress-responsive (MYB, MYC, LTR, etc.) elements indicate the significant functional role of *CDK*s and *CDKL*s in several abiotic stress responses and tolerance in peanut.

Innumerable developmental and physiological processes like shoot and root apical meristems formation, leaf and flower development, male gametogenesis, pollen wall formation, double fertilization, flowering time control, trichome development, plant immunity, drought and salinity tolerance are regulated by CDKs [11, 15–17, 19, 20, 22, 69–71]. In tomatoes, cell expansion in the developing fruit is strongly regulated by transcription of *SlPPC2* (PEPC; EC 4.1.1.31) gene and therefore, its promoter can be used for transcriptional regulation of cell cycle related genes [72]. Fruit-specific over-expression of *SlKRP1* (a CDK inhibitor) under SlPEPC2 promoter (ProPECPC2) decreased endoreduplication without altering fruit morphology and cell division activity [73]. Kip-related protein or *KRP2* overexpression in Arabidopsis stimulated endoreduplication by inhibiting mitotic cell cycle-specific CDKA;1 kinase complexes, which results in inhibition of plant growth by reducing cell cycle progression [74]. Transgenic Arabidopsis plant overexpressing tobacco CDK inhibitor or *NtKIS1a*, an interactor of CDK and D-cyclins, drastically reduced plant growth by inhibiting cell cycle progression [75]. On the other hand, overexpression of *CcCKS* (cyclin-dependent kinase regulatory subunit gene) gene of pigeonpea in Arabidopsis showed higher biomass and enhanced tolerance to salinity and drought stresses [76]. In our study, the *CDK* and *CDKL* genes showed a distinct pattern of expression in different tissues, suggesting that these genes have crucial roles in the growth and development of peanut (Fig. 8). Further correlation analysis revealed that the expression of various genes in the plant organs was significantly correlated; most genes were positively correlated; whilst some were negatively correlated, like *AhCDKG2c*-*AhCDKL25* (-0.53), *AhCDKL18*-*AhCDKL25* (-0.52), *AhCDKL22*-*AhCDKL24* (-0.47) (Fig. 9A). Moreover, a few duplicated genes showed diverse expression patterns in different tissues, suggesting their functional divergence. The duplicated *CDKB1* genes showed variable expressions in shoot and gynophore tips, and *AhCDKB1a* showed much higher expressions in these tissues relative to *AhCDKB1b*. The segmentally duplicated *CDKD* genes showed high expression variations in multiple tissues, including main stem leaf, shoot tips, gynophore tips, perianth, pod and multiple seed stages. In addition, *CDKE* gene pair displayed varying expression patterns in perianth, gynoecium, stalk and shoot tips. *AhCDKG2c/AhCDKG2d* gene pair also showed divergent expressions in leaf tissues (Fig. 9B). This diversity of expression patterns suggested a broad range of biological functions of *CDK* and *CDKL* genes in peanut.

Correlation analysis demonstrated the similarities and differences of *CDK* and *CDKL* gene’s expression across the 22 different tissues. As expected, strong positive correlations were observed among similar group CDK members, whereas certain unrelated *CDK* and *CDKL* gene pairs showed positive correlations suggesting their possible collective roles during plant development. In addition, clustering of genes based on the expression patterns in different tissue types, like tissues, root-nodules, floral organs, and pod-pericarp-seed tissues, identified the possible co-expressions (genes showing similar expression patterns) of different sets of *CDK* and *CDKL* genes in each of the four tissue sets and suggested that different *CDK* and *CDKL* gene combinations might regulate each development stage or tissue.

In the past decade, *CDK* and *CDKL* have been largely investigated in plants but no detailed analysis of expression levels of *CDK* and *CDKL* gene family members has been reported in response to abiotic stresses. These genes possessed multiple drought stress-responsive cis-acting elements, thus we investigated their expression levels in the presence of ABA, PEG and mannitol. We found that *CDK* and *CDKL* genes responded to drought stress. The expression of multiple *CDK* and *CDKL* genes was enhanced at early time point of ABA treatment but was declined after longer exposure to ABA, indicating that *CDK* and *CDKL* might have diverse molecular mechanisms to retain protection against different drought signals (Fig. 10). In particular, most *CDK* and *CDKL* genes were drastically downregulated in response to PEG and mannitol treatment, suggesting these abiotic stress signals might inhibit cell proliferation by decreasing the expression of *CDK* and *CDKL* genes (Fig. 11 and Fig. 12).

## Conclusions

This genome-wide study of CDK group of genes in peanuts and their progenitors identified 104 *CDK*s and *CDKL*s across the three *Arachis* species (*Arachis hypogaea*, *Arachis duranensis* and *Arachis ipaensis*). The comprehensive analysis of *CDK* and *CDKL* gene family provided important information such as gene architecture, conserved motifs, chromosomal localization, whole-genome duplications, syntenic relationships, selection pressures and promoter elements. These findings provide an insight into the molecular evolution of *CDK* and *CDKL* gene family in cultivated peanut from its progenitors. Analysis of available peanut transcriptomic data revealed the expression patterns of *CDK* and *CDKL* genes in different tissues and highlighted their relevance in tissue development. A significant decrease in the gene expression levels of most *CDK* and *CDKL*s in response to abiotic stresses suggested a possible inhibition of cell proliferation. Further analysis of the individual genes would provide an insight into their specific functional mechanisms. The results specified in this study would be further utilized to identify and analyze candidate genes paramount to organising resilience mechanisms and enhancing yield of cultivated peanuts.

## Methods

### Identification of CDK and CDKL proteins

CDK and CDK-like (CDKL) protein sequences of Arabidopsis were compiled from The Arabidopsis Information Resource (TAIR) database (https://www.arabidopsis.org/). A BLASTP analysis using the Arabidopsis CDK queries with default parameters was employed to identify the homologous amino acid sequences in *A. hypogaea* (cultivated peanut) and its diploid parents, *A. duranensis* and *A. ipaensis* from PeanutBase (https://www.peanutbase.org/blast)*.* The blast hits with zero E-value were only considered as significant. CDK and CDKL group classification of *Arabidopsis thaliana* facilitated the nomenclature of the putative CDK and CDKL proteins of *A. hypogaea*, *A. duranensis* and *A. ipaensis*. Significant physicochemical properties of the identified proteins such as the protein sequence length, molecular weight (M.W.) and theoretical isoelectric point (pI) were analysed using EMBOSS Pepstats (https://www.ebi.ac.uk/Tools/seqstats/emboss_pepstats/) [77]. The subcellular localization predictions were made using CELLO v.2.5 (http://cello.life.nctu.edu.tw/) [78].

### Multiple sequence alignment and phylogenetic analysis

The presumed CDK and CDKL protein sequences from *A. hypogaea, A. duranensis and A. ipaensis* were aligned with the *Arabidopsis* counterparts using Clustal Omega software with default parameters [79]. For the phylogenetic analysis, a Maximum Likelihood (ML) tree was generated using MEGA7 with the following parameters: Jones-Taylor-Thornton (JTT) model; uniform rates; 1000 bootstrap replicates [80].

### Gene structure and composition of CDK and CDKL protein motifs

Gene structures of the *A. hypogaea, A. duranensis and A. ipaensis* CDKs were mapped using the Gene Structure Display Server (http://gsds.gao-lab.org/) [81]. The conserved motifs in *Arachis* CDKs and CDKLs were identified using MEME (https://meme-suite.org/meme/tools/meme) [82] with the maximum number of motifs to be discovered set at 10.

### Chromosomal mapping, gene duplication and syntenic analysis

The positional information regarding the putative *CDK*s and *CDKL*s in the three *Arachis* species were procured from PeanutBase, and the mapping of the genes on the chromosomes with their relative distances was performed using MapChart software [83]. The Multiple Collinearity Scan toolkit (MCScanX) in TBtools with default parameters enabled the gene duplication study in *A. hypogaea* as well as the synteny analysis between *A. hypogaea* and its diploid parental genomes. The identified whole-genome/segmental duplicated genes and the syntenic maps between peanut and its progenitors were depicted using the R packages BioCircos (https://CRAN.R-project.org/package=BioCircos) and RIdeogram (https://CRAN.R-project.org/package=RIdeogram), respectively.

The CDK orthologous gene pairs between *A. hypogaea* and its diploid parents, as well as the paralogs or homoeologs in *A. hypogaea*, were identified by previously established criteria [84, 85]. Using the local BLAST analysis, the possible homolog pairs were determined if the alignment exceeded 85% length of the longer sequence, sequence identity surpassed 85% and had a zero E-value. Subsequently, the pairs of CDS sequences were aligned using MAFFT 7.0 and the synonymous (Ks) or non-synonymous (Ka) substitution rates were determined using DnaSP6 software [86]. The pairs with Ks < 0.01 and Ka/Ks ratio < 0.001 were discarded to maintain the validity.

### Cis-regulatory element analysis

The PlantCARE program identified cis-regulatory elements under default settings in the 1.5 kb upstream regions from the translational start sites of all the putative *AhCDKs*, *AdCDKs* and *AiCDKs* [87]. Apart from multiple types of cis-regulatory elements, this analysis mainly summarized the different types of cis-elements related to development, stress and phytohormone responses in each species.

### Gene expression analysis

Transcriptomic data depicting the *CDK* and *CDKL g*ene expressions in 22 tissues of different developmental stages were extracted from the Arachis eFP browser (http://bar.utoronto.ca/efp_arachis/cgi-bin/efpWeb.cgi). The FPKM values after normalization by log_2_ transformation were used to prepare the expression heatmap in MeV software. Further, expression-based gene cluster and gene–gene correlation analysis were performed using R packages [NbClust (https://cran.r-project.org/package=NbClust) and Hmisc (https://cran.r-project.org/web/package=Hmisc)].

### Plant materials, RNA extraction and real-time PCR

Two weeks old *A. hypogaea* seedlings (cultivar Kadiri 6) at four leaf stage were treated with 100 µM ABA, 20% PEG (polyethylene glycol) and 200 mM Mannitol for 1 h, 12 h and 24 h. Both untreated and treated samples were collected separately for RNA extraction and used for further qRT-PCR analysis. Total RNA was isolated using Trizol (Takara) as described previously [88]. Both oligo (dT) primers and reverse transcription system (Takara) were used for cDNA synthesis. Subsequently, quantitative RT-PCR reactions were performed using SYBR Green (Takara) in the Real-Time PCR System (Bio Rad) (Table S3). For internal normalization control, *Ubiquitin* gene was used as housekeeping gene.

## Supplementary Information

**Additional file 1:** **Table S1.** Physico chemical and subcellular localizations of CDKs and CDKLs.

**Additional file 2:** **Table S2.** Ka-Ks values of CDKs and CDKLs.

**Additional file 3**: **Table 3.** Primer sequences used in qRT-PCR analysis.

**Additional file 4:** **Table 4.** Gene IDs of *CDK* and *CDKL *genes used in expression analysis by qRT-PCR.

**Additional file 5:** **Fig. S1.** Multiple alignments of cyclin-binding domains of CDKs. **Fig. S2.** Multiple alignments of cyclin-binding domains of CDKLs. **Fig. S3.** Multiple alignments of T-loop regions of CDKs. **Fig. S4.** Multiple alignments of T-loop regions of CDKLs. **Fig. S5.** Phylogenetic tree and gene structure analysis of 52 CDKs and CDKLs of Arachis duranensis and Arachis ipaensis. **Fig. S6.** Ten identified conserved protein motifs in Arachis hypogaea. **Fig. S7.** Ten identified conserved protein motifs in Arachis duranensis. **Fig. S8.** Ten identified conserved protein motifs in Arachis ipaensis. **Fig. S9.** Phylogenetic tree and protein motif structure analysis of CDK and CDKL proteins of Arachis duranensis and Arachis ipaensis. **Fig. S10.** The distributions of CDK and CDKL genes of Arachis duranensis across 10 chromosomes. **Fig. S11.** The distributions of CDK and CDKL genes of Arachis ipaensis across 10 chromosomes. **Fig. S12.** The cis-elements distribution in the promoter of CDK and CDKL genes in Arachis duranensis. **Fig. S13.** The cis-elements distribution in the promoter of CDK and CDKL genes in Arachis ipaensis. **Fig. S14.** Percentage of promoter cis-elementsin cultivated peanut and its diploid parents.

## Data Availability

The datasets analysed during the current study are available in PeanutBase (https://www.peanutbase.org/blast).
