# Supplementary material for: Genome-wide identification, evolutionary and expression analysis of the cyclin-dependent kinase gene family in peanut
Source: BMC Plant Biol. 2023 Jan 19;23:43. doi: 10.1186/s12870-023-04045-w (PMC9850575; doi:10.1186/s12870-023-04045-w)
Supplement: Supplementary file 3 — Additional file 3: Table 3. Primer sequences used in qRT-PCR analysis. [file 12870_2023_4045_MOESM3_ESM.docx]

| **Gene** | **Primer** |
| --- | --- |
| AhCDKA1cFP | 5’ TGCACAGTGAGAAGCGATTG 3’ |
| AhCDKA1cRP | 5’ GTGCGGCGATCTATCAACAA 3’ |
| AhCDKB2aFP | 5’ AGGTCAGAACAAGGAAGGCA 3’ |
| AhCDKB2aRP | 5’ AGTCCCTGTGCAAGATTCCA 3’ |
| AhCDKC1aFP | 5’ GATCCATTGCCATGTGACCC 3’ |
| AhCDKC1aRP | 5’ TTGGTTAGGTCCTGATCGCA 3’ |
| AhCDKD1dFP | 5’ TCCTACCTCCAAATGACGCT 3’ |
| AhCDKD1dRP | 5’ CTGTACCACCGAGCAAACAC 3’ |
| AhCDKE1aFP | 5’ CAAACCAATCGCGGCAAATC 3’ |
| AhCDKE1aRP | 5’ ATGTCCGCGTGATTGATGTG 3’ |
| AhCDKF1bFP | 5’ GGAACTCGTTGGTTTAGGGC 3’ |
| AhCDKF1bRP | 5’ CATCGAGGTTGCCCAAAACA 3’ |
| AhCDKG2aFP | 5’ ATGAAAGACAGCGAGGTTGC 3’ |
| AhCDKG2aRP | 5’ GAGGTGGAAGAGCAGTCACT 3’ |
| AhCDKL5FP | 5’ CCGAGACATCAAGGGATCGA 3’ |
| AhCDKL5RP | 5’ AGCCAACACTCCAGAGATCC 3’ |
| AhCDKL27FP | 5’ GGCAAGTGTCCGATTCATGG 3’ |
| AhCDKL27RP | 5’ AGTGAACTTGACTCCAGGGG 3’ |

**Additional Table 3. Primer sequences used in qRT-PCR analysis.**
