# Supplementary material for: Genome-wide identification, evolutionary and expression analysis of the cyclin-dependent kinase gene family in peanut
Source: BMC Plant Biol. 2023 Jan 19;23:43. doi: 10.1186/s12870-023-04045-w (PMC9850575; doi:10.1186/s12870-023-04045-w)
Supplement: Supplementary file 4 — Additional file 4: Table 4. Gene IDs of CDK and CDKL genes used in expression analysis by qRT-PCR. [file 12870_2023_4045_MOESM4_ESM.docx]

**Additional Table 4. Gene IDs of *CDK* and *CDKL* genes used in expression analysis by qRT-PCR.**

| **Gene Name** | **Gene IDs** |
| --- | --- |
| ***AhCDKA1c*** | [**Arahy.XZ8R5P**](https://peanutbase.org/gbrowse_peanut1.0?query=h_feat=Query_1;q=Arahy.XZ8R5P;add=+BLAST+BlastHit=1..294;h_feat=BlastHit) |
| ***AhCDKB2a*** | [**Arahy.Q1AFG0**](https://peanutbase.org/gbrowse_peanut1.0?query=h_feat=Query_1;q=Arahy.Q1AFG0;add=+BLAST+BlastHit=17..321;h_feat=BlastHit) |
| ***AhCDKC1a*** | [**Arahy.IJVD3H**](https://peanutbase.org/gbrowse_peanut1.0?query=h_feat=Query_1;q=Arahy.IJVD3H;add=+BLAST+BlastHit=1..523;h_feat=BlastHit) |
| ***AhCDKD1d*** | [**Arahy.S5Z9V6**](https://peanutbase.org/gbrowse_peanut1.0?query=h_feat=Query_1;q=Arahy.S5Z9V6;add=+BLAST+BlastHit=8..397;h_feat=BlastHit) |
| ***AhCDKE1a*** | [**Arahy.LW25T7**](https://peanutbase.org/gbrowse_peanut1.0?query=h_feat=Query_1;q=Arahy.LW25T7;add=+BLAST+BlastHit=22..446;h_feat=BlastHit) |
| ***AhCDKF1b*** | [**Arahy.HT6INV**](https://peanutbase.org/gbrowse_peanut1.0?query=h_feat=Query_1;q=Arahy.HT6INV;add=+BLAST+BlastHit=4..440;h_feat=BlastHit) |
| ***AhCDKG2a*** | **Arahy.93GJX5** |
| ***AhCDKL*5** | **Arahy.XZ331S** |
| ***AhCDKL*27** | **Arahy.QF03JM** |
| ***AhUbiquitin*** | **NC_037631.1, Arahy.U26HEC** |
