# Supplementary material for: Genome-wide identification, evolutionary and expression analysis of the cyclin-dependent kinase gene family in peanut
Source: BMC Plant Biol. 2023 Jan 19;23:43. doi: 10.1186/s12870-023-04045-w (PMC9850575; doi:10.1186/s12870-023-04045-w)
Supplement: Supplementary file 5 — Additional file 5: Fig. S1. Multiple alignments of cyclin-binding domains of CDKs. Fig. S2. Multiple alignments of cyclin-binding domains of CDKLs. Fig. S3. Multiple alignments of T-loop regions of CDKs. Fig. S4. Multiple alignments of T-loop regions of CDKLs. Fig. S5. Phylogenetic tree and gene structure analysis of 52 CDKs and CDKLs of Arachis duranensis and Arachis ipaensis. Fig. S6. Ten identified conserved protein motifs in Arachis hypogaea. Fig. S7. Ten identified conserved protein motifs in Arachis duranensis. Fig. S8. Ten identified conserved protein motifs in Arachis ipaensis. Fig. S9. Phylogenetic tree and protein motif structure analysis of CDK and CDKL proteins of Arachis duranensis and Arachis ipaensis. Fig. S10. The distributions of CDK and CDKL genes of Arachis duranensis across 10 chromosomes. Fig. S11. The distributions of CDK and CDKL genes of Arachis ipaensis across 10 chromosomes. Fig. S12. The cis-elements distribution in the promoter of CDK and CDKL genes in Arachis duranensis. Fig. S13. The cis-elements distribution in the promoter of CDK and CDKL genes in Arachis ipaensis. Fig. S14. Percentage of promoter cis-elementsin cultivated peanut and its diploid parents. [file 12870_2023_4045_MOESM5_ESM.pdf]

```
CDKF1         -------------AFREIDALTILNGSPNVVVMHEY------------------------   80
AhCDKF1b      -------------AFREIDALQILQGSPNVVVLHEY------------------------   79
AhCDKF1a      -------------AFREIDALQILQGAPNVVVLHEY------------------------   79
AiCDKF1b      -------------AFREIDALQILQGAPNVVVLHEY------------------------   79
AdCDKF1       -------------AFREIDALQILQGSPNVVVLHEY------------------------   79
AiCDKF1a      -------------AFREIDALQILQGSPNVVVLHEY------------------------   83
CDKE1         ---------VSPTAIREIMLLREISHENVV-KLVNVH------------------------   92
AhCDKE1a      ---------VSPTAIREIMLLREITHENVV-KLVNVH------------------------   98
AhCDKE1b      ---------VSPTAIREIMLLREITHENVV-KLVNVH------------------------   98
AdCDKE1       ---------VSPTAIREIMLLREITHENVV-KLVNVH------------------------   98
AiCDKE1       ---------VSPTAIREIMLLREITHENVV-KLVNVH------------------------   98
CDKG1         ---------FPLTSLREINILLSCNHPAIV-NVKEVV------------------------   367
CDKG2         ---------FPLTSLREINILLSFHHPSIV-DVKEVV------------------------   472
AdCDKG2a      ---------FPLTSLREINILLSFHHPSIV-DVKEVV------------------------   473
AhCDKG2a      ---------FPLTSLREINILLSFHHPSIV-DVKEVV------------------------   473
AhCDKG2b      ---------FPLTSLREINILLSFHHPSIV-DVKEVV------------------------   473
AiCDKG2a      ---------FPLTSLREINILLSFHHPSIV-DVKEVV------------------------   518
AhCDKG2e      ---------FPLTSLREMNILLSFNHPSIV-DVKEVV------------------------   307
AhCDKG2f      ---------FPLTSLREMNILLSFNHPSIV-DVKEVV------------------------   307
AiCDKG2c      ---------FPLTSLREMNILLSFNHPSIV-DVKEVV------------------------   307
AdCDKG2c      ---------FPLTSLREMNILLSFNHPSIV-DVKEVV------------------------   307
AhCDKG2c      ---------FPLSSLREINILLSFDHPAIV-DVKEVV------------------------   338
AiCDKG2b      ---------FPLSSLREINILLSFDHPAIV-DVKEVV------------------------   338
AhCDKG2d      ---------FPLSSLREINILLSFDHPSIV-DVKEVV------------------------   339
AdCDKG2b      ---------FPLSSLREINILLSFDHPSIV-DVKEVV------------------------   339
AhCDKC1b      ILYRYFLSLFPITAIREIKILKKLHHENVIK-LKEIVT----------------SPGT--   108
AdCDKC1       ILYWYFLSLFPITAIREIKILKKLHHENVIK-LKEIVT----------------SPGT--   108
AhCDKC1a      ---------FPITAIREIKILKKLHHENVIK-LKEIVT----------------SPGP--   96
AiCDKC1       ---------FPITAIREIKILKKLHHENVIK-LKEIVT----------------SPD---   95
CDKC1         ---------FPITAIREIKILKKLHHENVIQ-LKEIVT----------------SPGR--   97
CDKC2         ---------FPITAIREIKILKKLHHENVIH-LKEIVT----------------SPGR--   97
CDKD1-1       ---------VNVTALREIKLLKELKHPHII-ELI--------------------------   74
CDKD1-3       ---------VNITALREIKMLKELKHPHII-LLI--------------------------   75
CDKD1-2       ---------VNFTALREIKLLKELNHPHIV-ELI--------------------------   76
AhCDKD1c      ---------VNFTALREIKLLKELKDPNII-ELI--------------------------   77
AiCDKD1a      ---------VNFTALREIKLLKELKDPNII-ELI--------------------------   77
AhCDKD1d      ---------VNFTALREIKLLKELKDPNII-ELI--------------------------   77
AdCDKD1b      ---------VNFTALREIKLLKELKDPNII-ELI--------------------------   77
AdCDKD1a      ---------VNFTALREIKLLKELKDPNII-ELI--------------------------   77
AiCDKD1b      ---------VNFTALREIKLLKELKDPNII-ELI--------------------------   77
AhCDKD1a      ---------VNFTALREIKLLKELKDPNII-ELI--------------------------   77
AhCDKD1b      ---------VNFTALREIKLLKELKDPNII-ELI--------------------------   77
CDKA1         ---------VPSTAIREISLLKEMQHSNI-VK----------------------------   65
AdCDKA1a      ---------VPSTAIREISLLKEMQHRNI-VRYTSAKCFLVIGYLIINEVDFLNANSKAI   93
AhCDKA1a      ---------VPSTAIREISLLKEMQHRNI-VR----------------------------   65
AhCDKA1d      ---------VPSTAIREISLLKEMQHRNI-VRYTSANCFLVIGYLIINEVDFLNANSKAI   93
AhCDKA1b      ---------VPSTAIREISLLKEMQHRNI-VR----------------------------   65
AhCDKA1c      ---------VPSTAIREISLLKEMQHRNI-VR----------------------------   65
AdCDKA1b      ---------VPSTAIREISLLKEMQHRNI-VR----------------------------   65
AiCDKA1       ---------VPSTAIREISLLKEMQHRNI-VR----------------------------   65
AhCDKB1a      ---------VPPTALREVSLLQMLSQSIYIVRLLSVE-----------------------   71
AdCDKB1       ---------VPPTALREVSLLQMLSQSIYIVRLLSVE-----------------------   71
AiCDKB1       ---------VPPTALREVSLLQMLSQSIYIVRLLSVE-----------------------   71
AhCDKB1b      ---------VPPTALREVSLLQMLSQSIYIVRLLSVE-----------------------   71
CDKB1-1       ---------IPPTALREISLLQMLSTSIYVVRLLCVE-----------------------   71
CDKB1-2       ---------IPPTALREISLLQMLSQSIYIVRLLCVE-----------------------   71
AhCDKB2a      ---------VPPTTLREVSILRMLSRDPHVVRLMDVK-----------------------   90
AdCDKB2       ---------VPPTTLREVSILRMLSRDPHVVRLMDVK-----------------------   90
AhCDKB2b      ---------VPPTTLREVSILRMLSRDPHVVRLMDVK-----------------------   90
AiCDKB2       ---------VPPTTLREVSILRMLSRDPHVVRLMDVK-----------------------   90
CDKB2-1       ---------VPSTTLREISILRMLARDPHVVRLMDVK-----------------------   81
CDKB2-2       ---------VPPTTLREISILRMLARDPHIVRLMDVK-----------------------   83
                           ::**:  *
```

Fig. S1. Multiple alignments of cyclin-binding domains of CDKs. Group-wise alignment of *Arachis hypogaea*, *Arachis duranensis*, *Arachis ipaensis* and *Arabidopsis thaliana* represent the cyclin-binding domains of CDKs. CDKA-PSTAIRE motif, CDKB1-PPTALRE motif, CDKB2-PPTTLRE motif, CDKC-PITAIRE motif, CDKD-NFTALRE motif, CDKE-SPTAIRE motif, CDKG-PLTSLRE motif. Specific cyclin-binding domain is absent in CDKFs. * indicates highly conserved residues among all groups of CDKs.

```
CDKL1       --------------------KIGQGTYSSVFRAREVETGKMVALKKVKFDNLQPESIRFMAREI    153
AhCDKL3     --------------------KIGQGTYSSVFQAREVHSGRMVALKKVRFDNFQPESIRFMAREI    206
AdCDKL2     --------------------KIGQGTYSSVFQAREVHSGRMVALKKVRFDNFQPESIRFMAREI    206
AhCDKL4     --------------------KIGQGTYSSVFQAREVHSGRMVALKKVRFDNFQPESIRFMAREI    206
AiCDKL2     --------------------KIGQGTYSSVFQAREVHSGRMVALKKVRFDNFQPESIRFMAREI    206
AdCDKL1     --------------------KIGQGTYSSVFRAREVETGRMFALKKVRFDNLQPESIRFMAREI    160
AhCDKL1     --------------------KIGQGTYSSVFRAREVETGRMFALKKVRFDNLQPESIRFMAREI    160
AhCDKL2     --------------------KIGQGTYSSVFRAREVETGRMFALKKVRFDNLQPESIRFMAREI    161
AiCDKL1     --------------------KIGQGTYSSVFRAREVETGRMFALKKVRFDNLQPESIRFMAREI    161
AiCDKL4     --------------------KIGQGTYSSVFRARELETGKIVALKKVRFDNFEPESVRFMAREI    155
AdCDKL4     --------------------KIGQGTYSSVFRARELETGKIVALKKVRFDNFEPESVRFMAREI    155
AhCDKL7     --------------------KIGQGTYSSVFRARELETGKIVALKKVRFDNFEPESVRFMAREI    155
AhCDKL8     --------------------KIGQGTYSSVFRARELETGKIVALKKVRFDNFEPESVRFMAREI    155
AdCDKL3     --------------------KIGQGTYSSVFRAKELETGKIVALKKVRFDNFEPESVRFMAREI    116
AiCDKL3     --------------------KIGQGTYSSVFRAKELETGKIVALKKVRFDNFEPESVRFMAREI    125
AhCDKL5     --------------------KIGQGTYSSVFRAKELETGKIVALKKVRFDNFEPESVRFMAREI    134
AhCDKL6     --------------------KIGQGTYSSVFRAKELETGKIVALKKVRFDNFEPESVRFMAREI    134
CDKL2       --------------------KIGQGTYSNVFRAVETETGKIVALKKVRFDNFEPESVKFMAREI    169
CDKL3       --------------------KIGQGTYSSVFRARETETGRIVALKKVRFDNFEPESVRFMAREI    179
CDKL7       --------------------KIGQGTYSNVYKAKDLLSGKIVALKKVRFDNLEAESVKFMAREI    162
AhCDKL16    --------------------KIGQGTYSNVYKARDLLSGKIVALKKVRFDNLEPESVKFMAREI    149
AiCDKL8     --------------------KIGQGTYSNVYKARDLLSGKIVALKKVRFDNLEPESVKFMAREI    149
AhCDKL17    --------------------KIGQGTYSNVYKARDLLSGKIVALKKVRFDNLEPESVKFMAREI    149
AdCDKL7     --------------------KIGQGTYSNVYKARDLLSGKIVALKKVRFDNLEPESVKFMAREI    149
AhCDKL24    --------------------KIGQGTYSNVYKARDLVSGKIVALKKVRFDDLEGESVKFMAREI    104
AiCDKL11    --------------------KIGQGTYSNVYKARDLVSGKIVALKKVRFDDLEGESVKFMAREI    104
AhCDKL25    --------------------KIGQGTYSNVYKARDLVSGKIVALKKVRFDHLEGESVKFMAREI    111
AdCDKL11    --------------------KIGQGTYSNVYKARDLVSGKIVALKKVRFDHLEGESVKFMAREI    111
CDKL8       --------------------KIGSGTYSNVYKAKDSLTGNIVALKKVRCDVNERESLKFMAREI    186
CDKL9       --------------------KIGQGTYSNVYKAKDMLTGKIVALKKVRFDNLEPESVKFMAREI    166
AhCDKL10    --------------------KIGQGTYSNVYKARDTLTGKIVALKKVRFDNLEPESVKFMAREI    161
AdCDKL6     --------------------KIGQGTYSNVYKARDTLTGKIVALKKVRFDNLEPESVKFMAREI    161
AhCDKL11    --------------------KIGQGTYSNVYKARDTLTGKIVALKKVRFDNLEPESVKFMAREI    160
AiCDKL6     --------------------KIGQGTYSNVYKARDTLTGKIVALKKVRFDNLEPESVKFMAREI    160
AiCDKL12    --------------------KIGQGTYSNVYKAKDMMTGKIVALKKVRFDNLEPESVKFMAREI    165
AdCDKL13    --------------------KIGQGTYSNVYKAKDMMTGKIVALKKVRFDNLEPESVKFMAREI    166
AhCDKL12    --------------------KIGQGTYSNVYKAKDMMTGKIVALKKVRFDNLEPESVKFMAREI    165
AhCDKL13    --------------------KIGQGTYSNVYKAKDMMTGKIVALKKVRFDNLEPESVKFMAREI    166
AhCDKL14    --------------------KIGQGTYSNVYKAKDMLTGKIVALKKVRFDNLEPESVKFMAREI    168
AiCDKL7     --------------------KIGQGTYSNVYKAKDMLTGKIVALKKVRFDNLEPESVKFMAREI    168
AhCDKL15    --------------------KIGQGTYSNVYKAKDMLTGKIVALKKVRFDNLEPESVKFMAREI    168
AdCDKL12    --------------------KIGQGTYSNVYKAKDMLTGKIVALKKVRFDNLEPESVKFMAREI    168
CDKL4       --------------------QIGGGTFSKVFKARDLLRNKTVALKRIRFDINNSESIKCIAREI    151
CDKL5       --------------------KIGQGTYSSVYKARDLTNNKIVALKRVRFDLSDLESVKFMAREI    185
CDKL6       --------------------KIGQGTYSSVYRARDLLHNKIVALKKVRFDLNDMESVKFMAREI    261
AhCDKL19    --------------------KIGQGTYSTVYKARDITHQKIVALKRVRFDNLDPESVKFMAREI    170
AhCDKL18    --------------------KIGQGTYSTVYKARDITQQKIVALKRVRFDNLDPESVKFMAREI    170
AdCDKL8     --------------------KIGQGTYSTVYKARDITQQKIVALKRVRFDNLDPESVKFMAREI    170
AiCDKL9     --------------------KIGQGTYSTVYKARDITHQKIVALKRVRFDNLDPESVKFMAREI    170
AhCDKL20    --------------------KIGQGTYSTVYKARDVTNQKIVALKKVHFDSLNPESIKFMAREI    182
AiCDKL14    --------------------KIGQGTYSTVYKARDVTNQKIVALKKVHFDSLNPESIKFMAREI    164
AhCDKL21    --------------------KIGQGTYSTVYKARDVTNQKIVALKKVYFDSLNPESIKFMAREI    182
AdCDKL10    --------------------KIGQGTYSTVYKARDVTNQKIVALKKVYFDSLNPESIKFMAREI    182
CDKL10      --------------------MIGQGTYSSVYRARDLETNQIVALKKVRFANMDPESVRFMAREI    194
CDKL11      --------------------KIGQGTYSSVYKARDLETNQLVALKKVRFANMDPDSVRFMAREI    211
AhCDKL22    -----------------DSKQIGQGTYSSVYRARDLETNKIVALKKVRFATMDPESVRFMAREI    155
AiCDKL10    -----------------DSKQIGQGTYSSVYRARDLETNKIVALKKVRFATMDPESVRFMAREI    155
AhCDKL26    --------------------------------ARDLETNKIVALKKVRFATMDPESVRFMAREI    149
AdCDKL9     ---------------------IGQGTYSSVYRARDLETNKIVALKKVRFATMDPESVRFMAREI    180
AhCDKL28    --------------------QIGQGAYSSVHKALDLESGKFVALKKVRFLSSDLASVRFMAREI    203
AdCDKL14    --------------------QIGQGAYSSVHKALDLESGKFVALKKVRFLSSDLASVRFMAREI    203
AhCDKL27    --------------------QIGQGAYSSVHKALDLESGKFVALKKVRFLSSDPASVRFMAREI    203
AiCDKL13    --------------------QIGQGAYSSVHKALDLESGKFVALKKVRFLSSDPASVRFMAREI    203
CDKL15      --------------------KIGQGTYSNVYRARDLDQKKIVALKKVRFDNLEPESVRFMAREI    182
AhCDKL23    VQYFAQISGILLMIDCEVLMKIGQGTYSNVYRARDLEQRKIVALKKVRFDNLEPESVRFMAREI    238
AhCDKL9     --------------------KIGQGTYSNVYRARDLEQRKIVALKKVRFDNLEPESVRFMAREI    179
AiCDKL5     --------------------KIGQGTYSNVYRARDLEQRKIVALKKVRFDNLEPESVRFMAREI    179
AdCDKL5     --------------------KIGQGTYSNVYRARDLEQRKIVALKKVRFDNLEPESVRFMAREI    179
CDKL12      --------------------KIGQGTYSSVYKARDLETGKIVAMKKVRFVNMDPESVRFMAREI    195
CDKL13      ----------------------------------------MVAMKKVRFVNMDPESVRFMAREI    24
CDKL14      --------------------KIGQGTYSIVYKARDLETGKIVAMKKVRFANMDPESVRFMAREI    189
                                                     .*:*::     :  *:: :****
```

Fig. S2. Multiple alignments of cyclin-binding domains of CDKLs. Group-wise alignment of *Arachis hypogaea*, *Arachis duranensis*, *Arachis ipaensis* and *Arabidopsis thaliana* represent the cyclin-binding domains of CDKLs. CDK-like (CDKL)- (V/I/L)(R/K)(FM/CI)AREI motif. * indicates highly conserved residues among all groups of CDKLs.

```
CDKA1          RVLHRDLKPQNLLIDRRTNSLKLADFGLARAFGIPVRTFTHEVVTLWYRAPEILLGSHHY 181
AdCDKA1a       RVLHRDLKPQNLLIDRSSNALKLADFGLARAFGIPVRTFTHEVVTLWYRAPEILLGSHHY 240
AhCDKA1a       RVLHRDLKPQNLLIDRSSNALKLADFGLARAFGIPVRTFTHEVVTLWYRAPEILLGSHHY 181
AhCDKA1d       RVLHRDLKPQNLLIDRSSNALKLADFGLARAFGIPVRTFTHEVVTLWYRAPEILLGSHHY 214
AhCDKA1b       RVLHRDLKPQNLLIDRRTNSLKLADFGLARAFGIPVRTFTHEVVTLWYRAPEILLGSRHY 181
AhCDKA1c       RVLHRDLKPQNLLIDRRTNSLKLADFGLARAFGIPVRTFTHEVVTLWYRAPEILLGSRHY 181
AdCDKA1b       RVLHRDLKPQNLLIDRRTNSL---------------RTFTHEVVTLWYRAPEILLGSRHY 166
AtCDKA1        R-----------------------------------------VVTLWYRAPEILLGSRHY 140
               *                                         ***************:**

AhCDKB1a       IADLGLGRAFTVPLKSYTHEIVTLWYRAPEVLLGTTHYSTGVDMWSVGCIFA-------- 213
AdCDKB1        IADLGLGRAFTVPLKSYTHEIVTLWYRAPEVLLGTTHYSTGVDMWSVGCIFA-------- 213
AtCDKB1        IADLGLGRAFTVPLKSYTHEIVTLWYRAPEVLLGTTHYSTGVDMWSVGCIFA-------- 213
AhCDKB1b       IADLGLGRAFTVPLKSYTHEIVTLWYRAPEVLLGTTHYSTGVDMWSVGCIFA-------- 213
CDKB1-1        IADLGLGRAFTVPLKSYTHEIVTLWYRAPEVLLGSTHYSTGVDMWSVGCIFA-------- 210
CDKB1-2        IADLGLSRAFTVPLKAYTHEIVTLWYRAPEVLLGSTHYSTAVDIWSVGCIFA-------- 212
AhCDKB2a       IADLGLARAFTVPIKKYTHEILTLWYRAPEVLLGATHYSMAVDMWSVGCIFA-------- 222
AdCDKB2        IADLGLARAFTVPIKKYTHEILTLWYRAPEVLLGATHYSMAVDMWSVGCIFA-------- 222
AhCDKB2b       IADLGLARAFTVPIKKYTHEILTLWYRAPEVLLGATHYSMAVDMWSVGCIFA-------- 222
AtCDKB2        IADLGLARAFTVPIKKYTHEILTLWYRAPEVLLGATHYSMAVDMWSVGCIFGKDLVSSTK 230
CDKB2-1        IADLGLARAFTLPMKKYTHEILTLWYRAPEVLLGATHYSTAVDMWSVGCIFA-------- 213
CDKB2-2        IADLGLARAFTLPMKKYTHEILTLWYRAPEVLLGATHYSTGVDMWSVGCIFA-------- 215
               ******.****:*:* *****:************:**** .**:*******.

AhCDKC1a       LHRDIKGSNLLIDNEGNLKLADFGLARSFSNDHNANLTNRVITLWYRPPELLLGTTKYGP 227
AdCDKC1        LHRDIKGSNLLIDNEGNLKLADFGLARSFSNDHNANLTNRVITLWYRPPELLLGTTKYGP 234
AhCDKC1b       LHRDIKGSNLLIDNEGNLKLADFGLARSFSNDHNANLTNRVITLWYRPPELLLGTTKYGP 234
AtCDKC1        LHRDIKGSNLLIDNEGNLKLADFGLARSFSNDHNANLTNRVITLWYRPPELLLGTTKYGP 209
CDKC1          LHRDIKGSNLLIDNEGNLKLADFGLARSYSHDHTGNLTNRVITLWYRPPELLLGATKYGP 220
CDKC2          LHRDIKGSNLLIDNEGNLKLADFGLARSYSHDHTGNLTNRVITLWYRPPELLLGATKYGP 220
               ****************************:*:**..*******************:*****

CDKE1          MGDAEEHGIVKIADFGLARIYQAPLKPLSDNGVVVTIWYRAPELLLGSKHYTSAVDMWAV 222
AhCDKE1a       MGEGEEHGVVKIADFGLARIYQAPLKPLSENGVVVTIWYRAPELLLGAKHYTSAVDMWAV 228
AhCDKE1b       MGEGEEHGVVKIADFGLARIYQAPLKPLSENGVVVTIWYRAPELLLGAKHYTSAVDMWAV 228
AdCDKE1        MGEGEEHGVVKIADFGLARIYQAPLKPLSENGVVVTIWYRAPELLLGAKHYTSAVDMWAV 240
AtCDKE1        MGEGEEHGVVKIADFGLARIYQSPLKPLSENGVVVTIWYRAPELLLGAKHYTSAVDMWAV 228
               **:.****:*************:******:*****************:************

CDKG1          YLHTNWIIHRDLKPSNLLMNNCGELKICDFGMARQYGSPIKPYTQMVITQWYRPPELLLG 475
CDKG2          YLHDNWVLHRDLKTSNLLLNNRGELKICDFGLARQYGSPLKPYTHLVVTLWYRAPELLLG 579
AdCDKG2a       YLHDNWVLHRDLKTSNLLLNNRGELKICDFGLARQYGSPLKPYTHLVVTLWYRAPELLLG 580
AhCDKG2a       YLHDNWVLHRDLKTSNLLLNNRGELKICDFGLARQYGSPLKPYTHLVVTLWYRAPELLLG 580
AhCDKG2b       YLHDNWVLHRDLKTSNLLLNNRGELKICDFGLARQYGSPLKPYTHLVVTLWYRAPELLLG 580
AtCDKG2a       YLHDNWVLHRDLKTSNLLLNNRGELKICDFGLARQYGSPLKPYTHLVVTLWYRAPELLLG 625
AhCDKG2e       YLHDNWVLHRDLKSSNILLNKKGELKICDFGLSRQYGSPLKPYTPLVVTLRYRAPELLLG 413
AhCDKG2f       YLHDNWVLHRDLKSSNILLNKKGELKICDFGLSRQYGSPLKPYTPLVVTLRYRAPELLLG 413
AtCDKG2c       YLHDNWVLHRDLKSSNILLNKKGELKICDFGLSRQYGSPLKPYTPLVVTLRYRAPELLLG 413
AdCDKG2c       YLHDNWVLHRDLKSSNILLNNKGELKICDFGLSRQYGSPLKPYTPLVVTLRYRAPELLLG 413
AhCDKG2c       YLHDNWVLHRDLKTSNILLSDKGKLKICDFGLSRQFGSPLKPYTPIVVTLWYRAPELLLG 444
AtCDKG2b       YLHDNWVLHRDLKTSNILLSDKGKLKICDFGLSRQFGSPLKPYTPIVVTLWYRAPELLLG 444
AhCDKG2d       YLHDNWVLHRDLKTSNILLNDKGELKICDFGLSRQFGSPLKPYTPIVVTLWYRAPELLLG 445
AdCDKG2b       YLHDNWVLHRDLKTSNILLNDKGELKICDFGLSRQFGSPLKPYTPIVVTLWYRAPELLLG 445
               *** **::***** **:*:.. *:*******::**:***:**** :*:*  ** ******

CDKD1-1        LKGLEYCHGKWVLHRDMKPNNLLIGPNGQLKLADFGLARIFGSPGRKFTHQVFARWYRAPELLF     181
CDKD1-3        FKGLAYCHDKWVLHRDMKPNNLLIGVDGQLKLADFGLARIFGSPNRKFTHQVFARWYRAPELLF     182
CDKD1-2        LKGLAYCHKKWVLHRDMKPNNLLIGENGLLKLADFGLARLFGSPNRRFTHQVFATWYRAPELLF     183
AhCDKD1c       LKGLAFCHKKWVLHRDMKPNNLLIGSHGQLKLADFGLARIFGSPDRRFTHQVFARWYRAPELLF     184
AtCDKD1a       LKGLAFCHKKWVLHRDMKPNNLLIGSHGQLKLADFGLARIFGSPDRRFTHQVFARWYRAPELLF     184
AhCDKD1d       LKGLAFCHKKWVLHRDMKPNNLLIGSHGQLKLADFGLARIFGSPDRRFTHQVFARWYRAPELLF     184
AdCDKD1b       LKGLAFCHKKWVLHRDMKPNNLLIGSHGQLKLADFGLARIFGSPDRRFTHQVFARWYRAPELLF     184
AdCDKD1a       LKGLAVCHKKWVLHRDMKPNNLLIGSNGQLKLADFGLARIFGSPDRRFTHQVFARWYRAPELLF     184
AhCDKD1a       LKGLAVCHKKWVLHRDMKPNNLLIGSNGQLKLADFGLARIFGSPDRRFTHQVFARWYRAPELLF     184
AtCDKD1b       LKGLAVCHKKWVLHRDMKPNNLLIGSNGQLKLADFGLARIFGSPDRRFTHQVFARWYRAPELLF     184
               :***  ** **************** .* **********:****.*:******* *********

CDKF1          VVRDDTDKDSNVHDGDISCLATCTVSEMDDDLGRNSFSYDADEAVDDTQGLMTSCVGTRNFRPPELL 304
AhCDKF1b       S-AEDIDKDTNIPDGNTSCLATCTTSDIDDDPLKSSFSYEAREDDGEEAGCLTSCVGTRNFRAPELL 281
AtCDKF1a       S-ADDIDKDTNIPDGNTSCLATCTTSDIDDDPLKSSFTYEAREDEGEESGCLTSCVGTRNFRAPELL 285
AtCDKF1b       S-ADDIDKDTNIPDGNTSCLATCTTSDIDDDPLKSSFSYEAREDEGEESGCLTSCVGTRNFRAPELL 281
AhCDKF1a       S-ADDIDKDTNIPDGNTSCLATCTTSDIDDDPLKSSFSYEAREDEGEESGCLTSCVGTRNFRAPELL 281
AdCDKF1        S-AEDIDKDTNIPDGNTSCLATCTTSDIDDDPLKSSFSYEAREDEGEEAGCLTSCVGTRNFRAPELL 281
                  :* ***:*: **: *******.*::***  :.**:*:* *   .:   * :********** ****
```

Fig. S3. Multiple alignments of T-loop regions of CDKs. Group-wise alignment of *Arachis hypogaea*, *Arachis duranensis*, *Arachis ipaensis* and *Arabidopsis thaliana* represent the T-loop regions of CDKs. Black line indicates T-loop region of each CDK group. Probable phosphorylated threonine residues are represented by red colour. * indicates highly conserved residues among all groups of CDKs.

```
CDKL1            VLKLGDFGLANVVTP--SNKNQLTSRVVTLWYRAPELLMGSTSYGVSVDLWSVGCVFAEI 299
AhCDKL3          VLKIGDFGLANTLSP--NNKNPLTSRVVTLWYRAPELLMGSTNYGVSVDLWSVGCVFAEL 352
AdCDKL2          VLKIGDFGLANTLSP--NNKNPLTSRVVTLWYRAPELLMGSTNYGVSVDLWSVGCVFAEL 352
AhCDKL4          VLKIGDFGLANTLSP--NNKNPLTSRVVTLWYRAPELLMGSTNYGVSVDLWSVGCVFAEL 352
AiCDKL2          VLKIGDFGLANTLSP--NNKNPLTSRVVTLWYRAPELLMGSTNYGVSVDLWSVGCVFAEL 352
AdCDKL1          ILKIGDFGLANTISP--NNKHQLTSRVVTLWYRPPELLMGSTNYGVSVDLWSVGCVFAEL 310
AhCDKL1          ILKIGDFGLANTISP--NNKHQLTSRVVTLWYRPPELLMGSTNYGVSVDLWSVGCVFAEL 306
AhCDKL2          ILKIGDFGLANTISP--NNKHQLTSRVVTLWYRPPELLMGSTNYGVSVDLWSVGCVFAEL 307
AiCDKL1          ILKIGDFGLANTISP--NNKHQLTSRVVTLWYRPPELLMGSTNYGVSVDLWSVGCVFAEL 307
AiCDKL4          ILKVADFGLANFSSS-A-YRHPLTSRVVTLWYRPPELLLGSTDYGPSVDLWSVGCVFAEL 328
AdCDKL4          ILKVADFGLANFSSS-A-YRHPLTSRVVTLWYRPPELLLGSTDYGPSVDLWSVGCVFAEL 328
AhCDKL7          ILKVADFGLANFSSS-A-YRHPLTSRVVTLWYRPPELLLGSTDYGPSVDLWSVGCVFAEL 319
AhCDKL8          ILKVADFGLANFSSS-A-YRHPLTSRVVTLWYRPPELLLGSTDYGPSVDLWSVGCVFAEL 319
AdCDKL3          ILKVADFGLANWCNS-GN-KQPLTSRVVTLWYRPPELLLGSTDYSPSVDLWSVGCVFAEL 262
AiCDKL3          ILKVADFGLANWCNS-GNNKQPLTSRVVTLWYRPPELLLGSTDYSPSVDLWSVGCVFAEL 272
AhCDKL5          ILKVADFGLANWCNS-GNNKQPLTSRVVTLWYRPPELLLGSTDYSPSVDLWSVGCVFAEL 281
AhCDKL6          ILKVADFGLANWCNS-GNNKQPLTSRVVTLWYRPPELLLGSTDYSPSVDLWSVGCVFAEL 281
CDKL2            ILKVADFGLANFSNSSGHKKKPLTSRVVTLWYRPPELLLGATDYGASVDLWSVGCVFAEL 317
CDKL3            ILKVADFGLANFCNASGN-KQPLTSRVVTLWYRPPELLLGATEYGASVDLWSVGCVFAEL 326
CDKL7            ILKIADFGLATFYDP--KQKQTMTSRVVTLWYRPPELLLGATSYGTGVDLWSAGCIMAEL 308
AhCDKL16         ILKIADFGLATFFDP--KQKQVLTSRVVTLWYRPPELLLGATFYGVGVDLWSAGCILAEL 295
AiCDKL8          ILKIADFGLATFFDP--KQKQVLTSRVVTLWYRPPELLLGATFYGVGVDLWSAGCILAEL 295
AhCDKL17         ILKIADFGLATFFDP--KQKQVLTSRVVTLWYRPPELLLGATFYGVGVDLWSAGCILAEL 295
AdCDKL7          ILKIADFGLATFFDP--KQKQVLTSRVVTLWYRPPELLLGATFYGVGVDLWSAGCILAEL 295
AhCDKL24         ILKIADFGLAAFYDS--KQTRPMTNRVVTLWYRPPELLLGATFYGVGIDLWSAGCIFAEL 250
AiCDKL11         ILKIADFGLAAFYDS--KQTRPMTNRVVTLWYRPPELLLGATFYGVGIDLWSAGCIFAEL 250
AhCDKL25         ILKIADFGLAAFYDS--KQTRPMTNRVVTLWYRPPELLLGATFYGVGIDLWSAGCIFAEL 257
AdCDKL11         ILKIADFGLAAFYDS--KQTRPMTNRVVTLWYRPPELLLGATFYGVGIDLWSAGCIFAEL 257
CDKL8            VLRIGDFGLATFFDA--SKRQEMTNRVVTLWYRSPELLHGVVEYSVGVDLWSAGCILAEL 332
CDKL9            VLKIADFGLATIFDP--NHKRPMTSRVVTLWYRAPELLLGATDYGVGIDLWSAGCILAEL 312
AhCDKL10         VLKIADFGLASFFDP--HHKHPMTSRVVTLWYRPPELLLGATEYGVGVDLWSAGCILAEL 307
AdCDKL6          VLKIADFGLASFFDP--HHKHPMTSRVVTLWYRPPELLLGATEYGVGVDLWSAGCILAEL 307
AhCDKL11         VLKIADFGLASFFDP--HHKHPMTSRVVTLWYRPPELLLGATEYGVGVDLWSAGCILAEL 306
AiCDKL6          VLKIADFGLASFFDP--HHKHPMTSRVVTLWYRPPELLLGATEYGVGVDLWSAGCILAEL 306
AiCDKL12         ILKIADFGLASLFDP--NHKHPMTSRVVTLWYRPPELLLGATDYGVGIDLWSAGCILGEL 291
AdCDKL13         ILKIADFGLASLFDP--NHKHPMTSRVVTLWYRPPELLLGATDYGVGIDLWSAGCILGEL 280
AhCDKL12         ILKIADFGLASLFDP--NHKHPMTSRVVTLWYRPPELLLGATDYGVGIDLWSAGCILGEL 311
AhCDKL13         ILKIADFGLASLFDP--NHKHPMTSRVVTLWYRPPELLLGATDYGVGIDLWSAGCILGEL 312
AhCDKL14         VLKIADFGLASFFDP--NRKQPMTNRVVTLWYRPPELLLGATDYGVGIDLWSAGCILGEL 314
AiCDKL7          VLKIADFGLASFFDP--NRKQPMTNRVVTLWYRPPELLLGATDYGVGIDLWSAGCILGEL 314
AhCDKL15         VLKIADFGLASFFDP--NRKQPMTNRVVTLWYRPPELLLGATDYGVGIDLWSAGCILGEL 314
AdCDKL12         VLKIADFGLASFFDP--NRKQPMTNRVVTLWYRPPELLLGATDYGVGIDLWSAGCILGEL 314
CDKL4            VLKIADFGLATFFDP--HNSVPLTTHVATLWYRPPELLLGASHYGIGVDLWSTGCVIGEL 298
CDKL5            VLKIADFGLATFFDP--QNCVPLTSRVVTLWYRPPELLLGACHYGVGVDLWSTGCILGEL 331
CDKL6            VLKIADFGLATFFDP--AKSVSLTSHVVTLWYRPPELLLGASHYGVGVDLWSTGCILGEL 407
AhCDKL19         ILKIADFGLANFLDP--HLNVPLTSRV------TPELLLGASRYGVAVDLWSTGCILGEL 310
AhCDKL18         ILKIADFGLANFLDP--HHNVPLTSRVVTLWYRPPELLLGASHYGVAVDLWSTGCILGEL 316
AdCDKL8          ILKIADFGLANFLDP--HHNVPLTSRVVTLWYRPPELLLGASHYGVAVDLWSTGCILGEL 316
AiCDKL9          ILKIADFGLANFLDP--HLNVPLTSRVVTLWYRPPELLLGASRYGVAVDLWSTGCILGEL 316
AhCDKL20         VLKIADFGLASFFDP--HHSIPLTRRVVTLWYRPPELLLGANHYGVAVDLWSTGCILGEL 328
AiCDKL14         VLKIADFGLASFFDP--HHSIPLTRRVVTLWYRPPELLLGANHYGVAVDLWSTGCILGEL 310
AhCDKL21         VLKIADFGLASFFDP--HHSIPLTSRVVTLWYRPPELLLGANHYGVAVDLWSTGCILGEL 328
AdCDKL10         VLKIADFGLASFFDP--HHSIPLTSRVVTLWYRPPELLLGANHYGVAVDLWSTGCILGEL 328
CDKL10           NLKIGDFGLSNFYRG--QRKQPLTSRVVTLWYRPPELLLGSTDYGVTVDLWSTGCILAEL 340
CDKL11           NLKIGDFGLANFYQG--HQKQPLTSRVVTLWYRPPELLLGSTDYGVTVDLWSTGCILAEL 357
AhCDKL22         RLKIGDFGLATSFHP--SKGKPLTSRVVTLWYRPPELLLGATDYGVSVDLWSSGCILAEL 301
AiCDKL10         RLKIGDFGLATSFHP--SKGKPLTSRVVTLWYRPPELLLGATDYGVSVDLWSSGCILAEL 301
AhCDKL26         -------------QP--SKGKPLTSRVVTLWYRPPELLLGATDYGVSVDLWSSGCILAEL 245
AdCDKL9          RLKIGDFGLATSFQP--SKGKPLTSRVVTLWYRPPELLLGATDYGVSVDLWSSGCILAEL 326
AhCDKL28         NLKIGDFGLATVYDP--EKNQQLTSRVVTLWYRAPELLLGATEYGASIDMWSAGCILAEL 349
AdCDKL14         NLKIGDFGLATVYDP--EKNQQLTSRVVTLWYRAPELLLGATEYGAAIDMWSAGCILAEL 349
AhCDKL27         NLKIGDFGLATVYDP--EKNQQLTSRVVTLWYRAPELLLGATEYGAAIDMWSAGCILAEL 349
AiCDKL13         NLKIGDFGLATVYDP--EKNQQLTSRVVTLWYRAPELLLGATEYGAAIDMWSAGCILAEL 349
CDKL15           VLKIADFGLASFFDP--RQTQPLTSRVVTLWYRPPELLLGATRYGAAVDLWSAGCILAEL 328
AhCDKL23         ILKIADFGLASFFDP--NQIQPLTSRVVTLWYRPPELLLGATYYGTAVDLWSTGCILAEL 384
AhCDKL9          ILKIADFGLASFFDP--NQIQPLTSRVVTLWYRPPELLLGATYYGTAVDLWSTGCILAEL 325
AiCDKL5          ILKIADFGLASFFDP--NQIQPLTSRVVTLWYRPPELLLGATYYGTAVDLWSTGCILAEL 325
AdCDKL5          ILKIADFGLASFFDP--NQIQPLTSRVVTLWYRPPELLLGATYYGTAVDLWSTGCILAEL 325
CDKL12           VLKIGDFGLANFYRG--DGDLQLTSRVVTLWYRAPELLLGATEYGPAIDLWSAGCILTEL 341
CDKL13           VLKIGDFGLANIYHP--EQDQPLTSRVVTLWYRAPELLLGATEYGPGIDLWSVGCILTEL 170
CDKL14           VLKIGDFGLASFYKP--DQDQPLTSRVVTLWYRAPELLLGSTEYGPAIDLWSVGCILAEL 335
                                       :* :*       **** *   *.  :*:** **:: *:
```

Fig. S4. Multiple alignments of T-loop regions of CDKLs. Group-wise alignment of *Arachis hypogaea*, *Arachis duranensis*, *Arachis ipaensis* and *Arabidopsis thaliana* represent the T-loop regions of CDKLs. Black line indicates T-loop region of each CDK group. * indicates highly conserved residues among all groups of CDKLs.

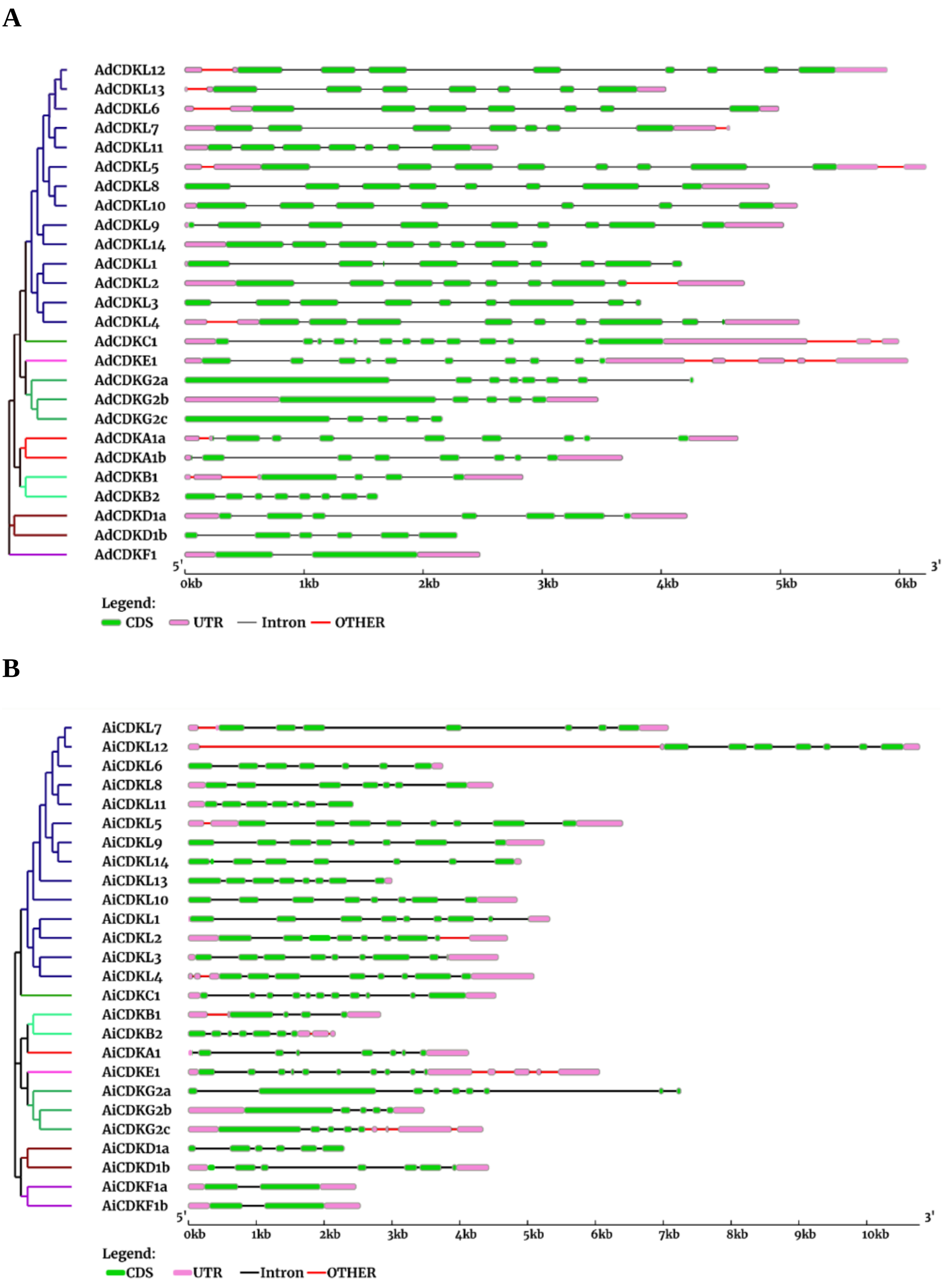

Fig. S5. Phylogenetic tree and gene structure analysis of 52 CDKs and CDKLs of *Arachis duranensis and Arachis ipaensis*. The phylogenetic tree was constructed using full-length amino acid sequences of CDKs and CDKLs proteins of *A. duranensis* **(A)** and *A. ipaensis* **(B)** using MEGA 7.0 with 1000 bootstrap replicates. Different colored lines indicate diverse groups. The exon-intron architecture highlights exons as green boxes and introns as black lines and magenta color box represents both 5' and 3' UTR regions.

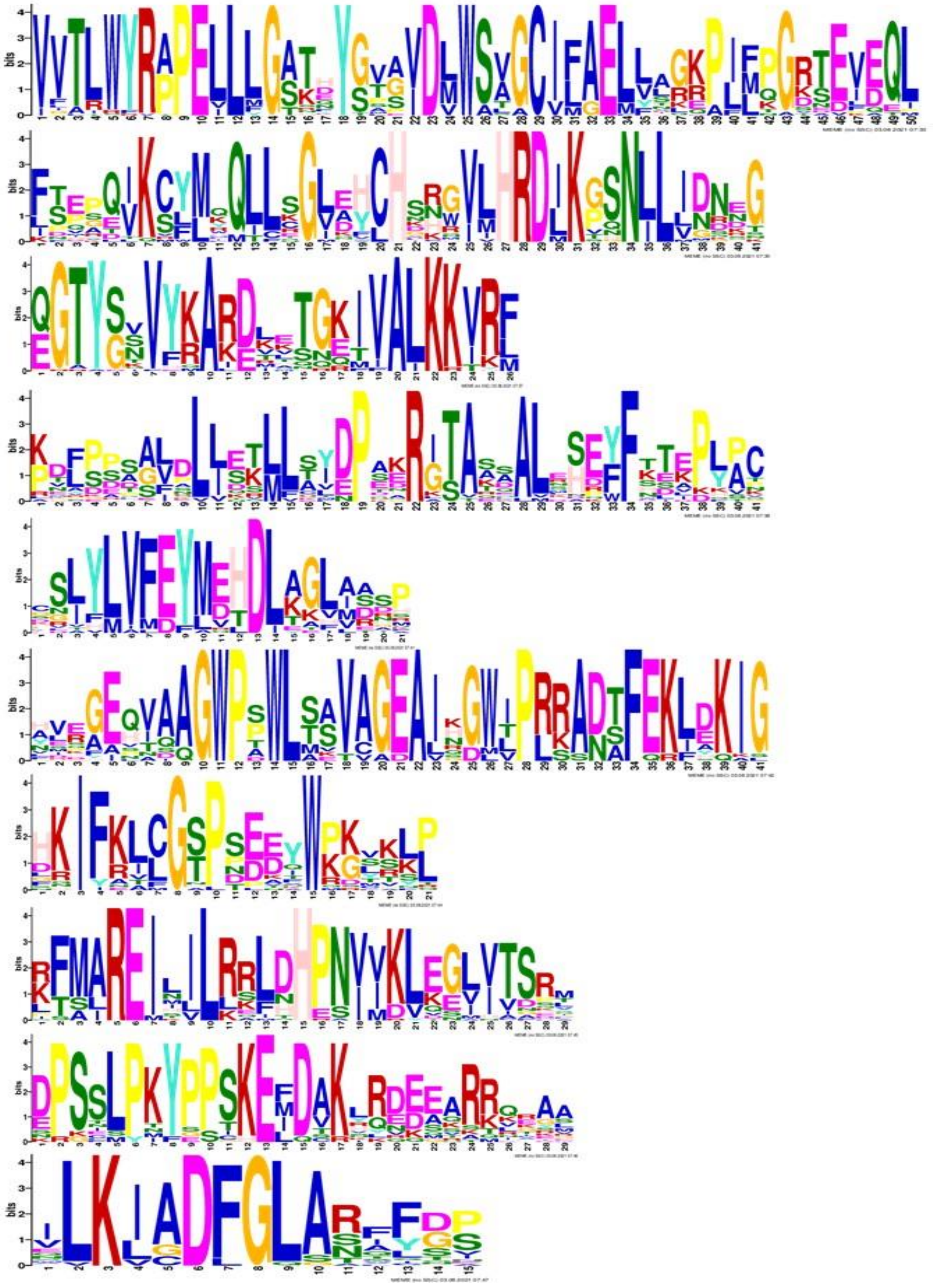

Fig. S6. Ten identified conserved protein motifs in *Arachis hypogaea*.

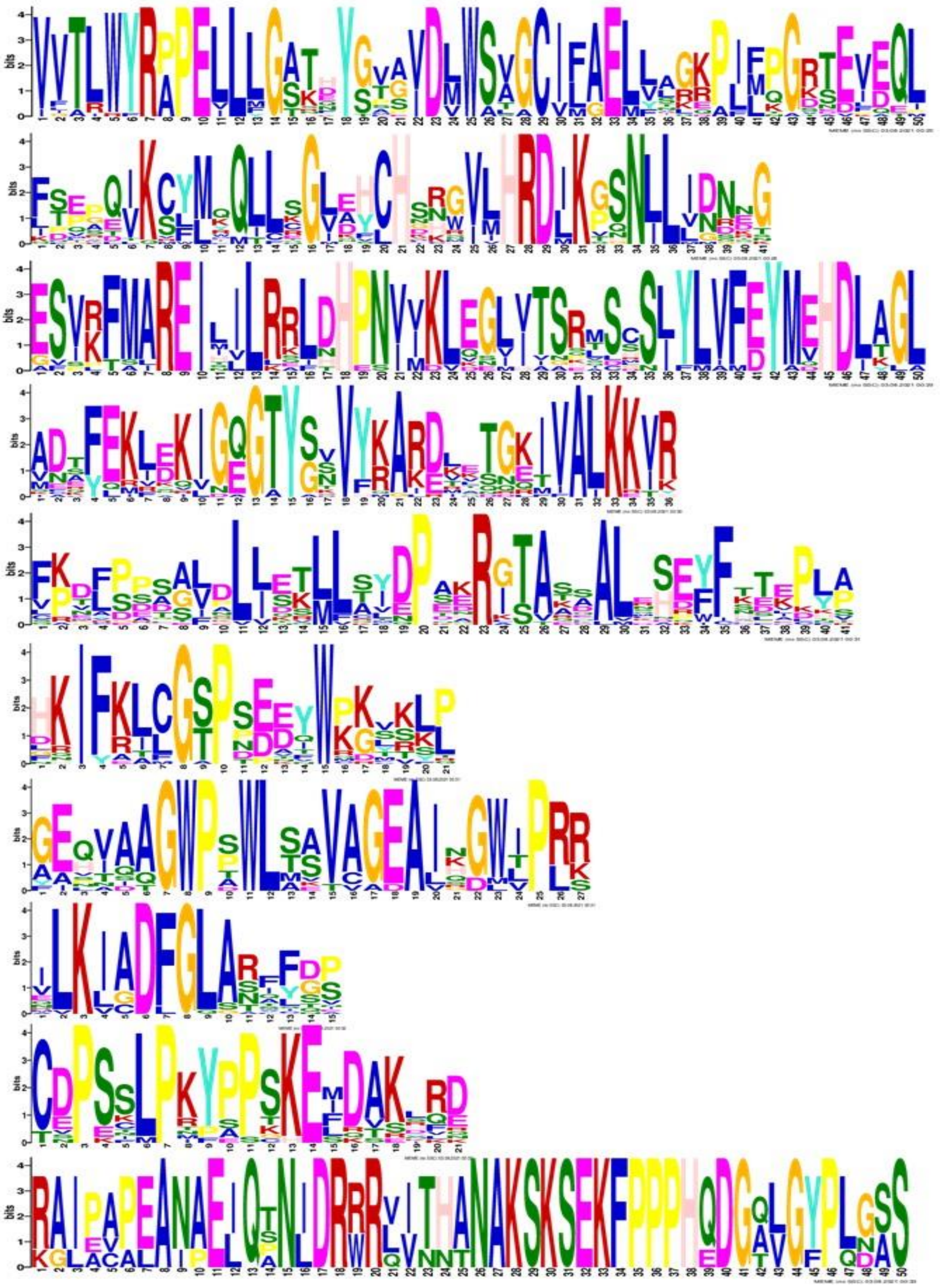

Fig. S7. Ten identified conserved protein motifs in *Arachis duranensis*.

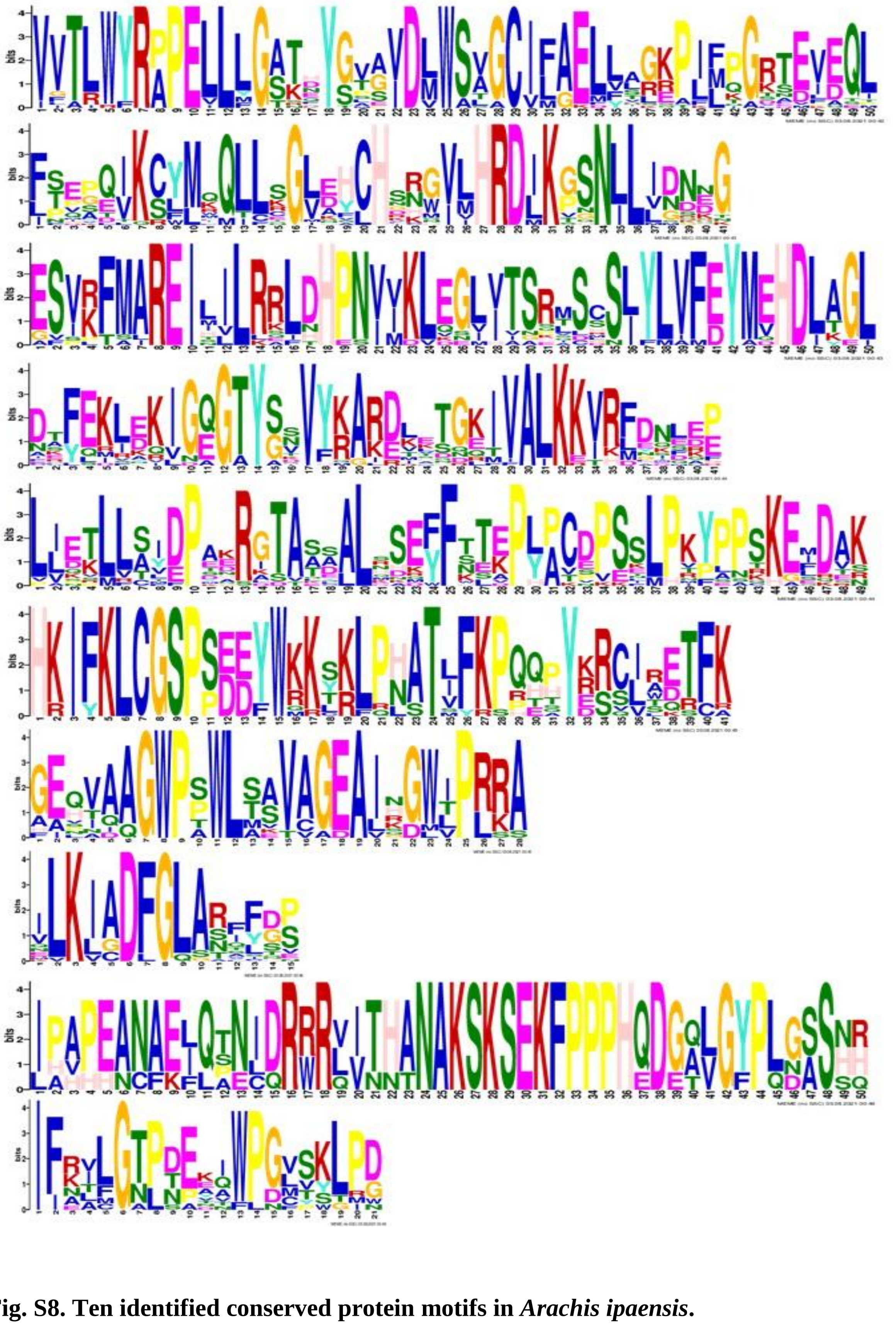

Fig. S8. Ten identified conserved protein motifs in *Arachis ipaensis*.

A

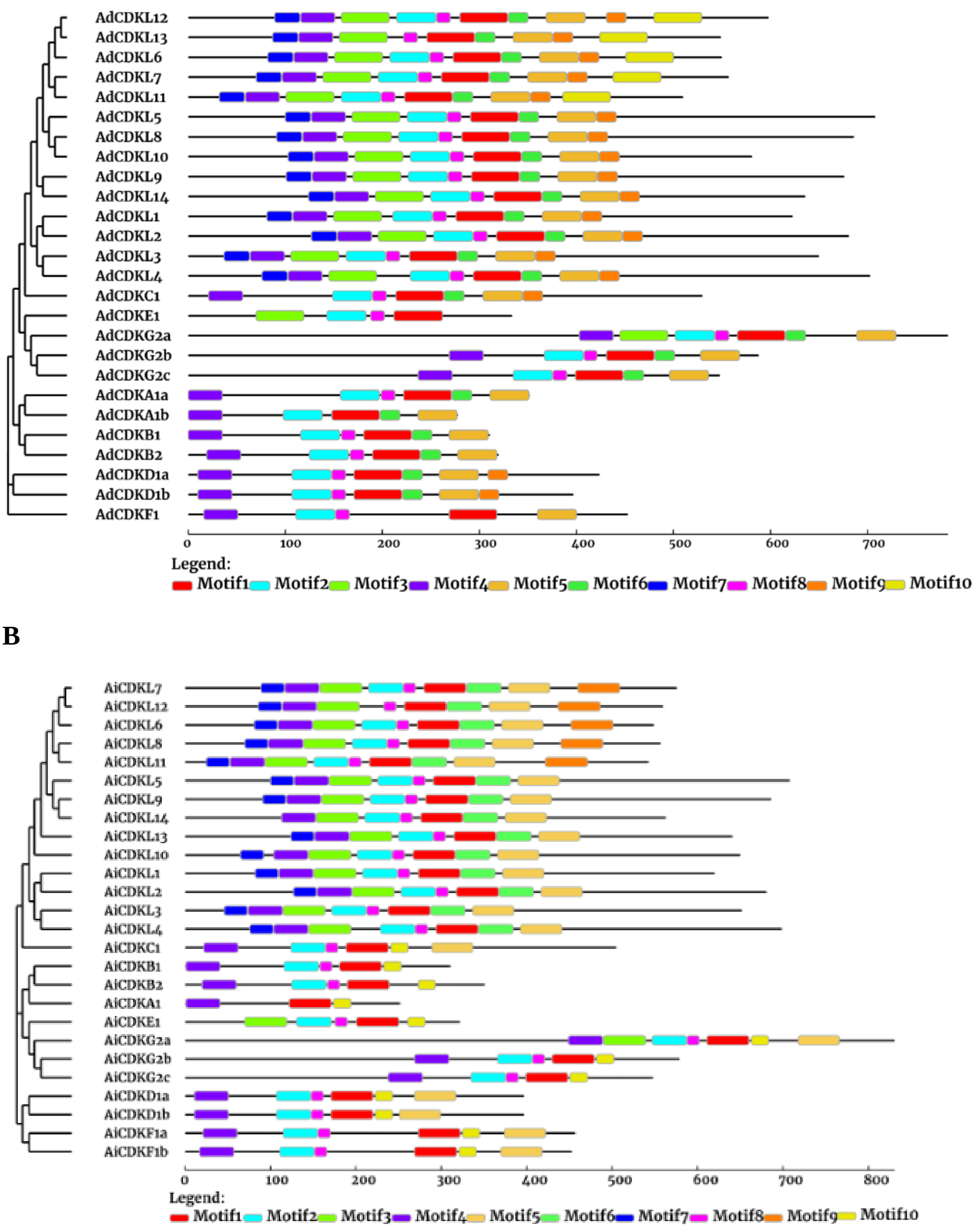

Fig. S9. Phylogenetic tree and protein motif structure analysis of CDK and CDKL proteins of *Arachis duranensis* and *Arachis ipaensis*. The phylogenetic tree was constructed using full-length amino acid sequences of CDKs and CDKLs proteins of *A. duranensis* **(A)** and *A. ipaensis* **(B)** using MEGA 7.0 with 1000 bootstrap replicates. The identified protein motifs are represented by colored boxes (Motif 1-Motif 10). Black lines indicate relative protein lengths.

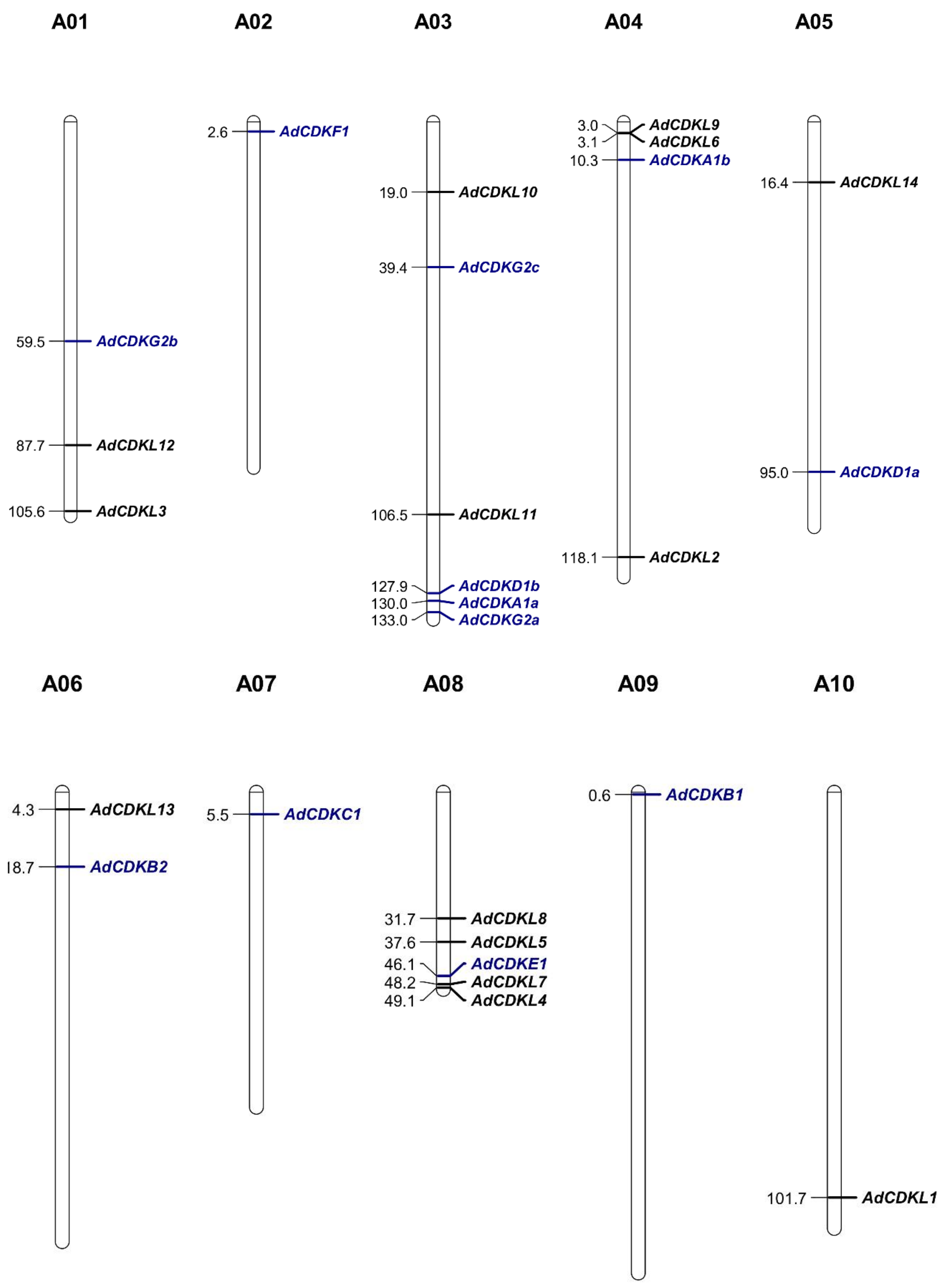

Fig. S10. The distributions of *CDK* and *CDKL* genes of *Arachis duranensis* across 10 chromosomes. The *CDKA-CDKG* group genes are depicted in blue, whereas the *CDKL* genes are highlighted in black. The values represent the relative start positions (Mbp) of each gene.

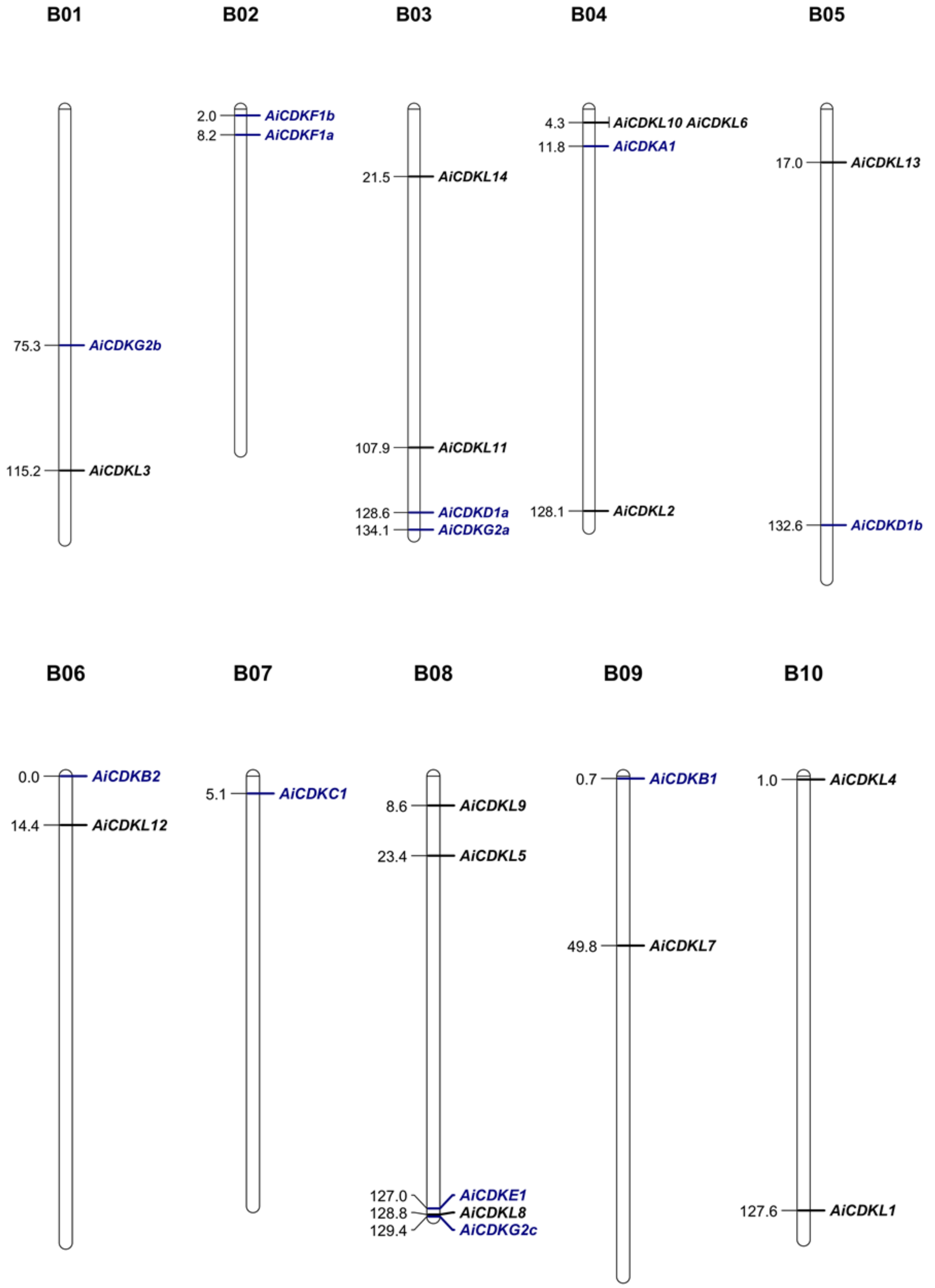

Fig. S11. The distributions of *CDK* and *CDKL* genes of *Arachis ipaensis* across 10 chromosomes. The *CDKA-CDKG* group genes are depicted in blue, whereas the *CDKL* genes are highlighted in black. The values represent the relative start positions (Mbp) of each gene.

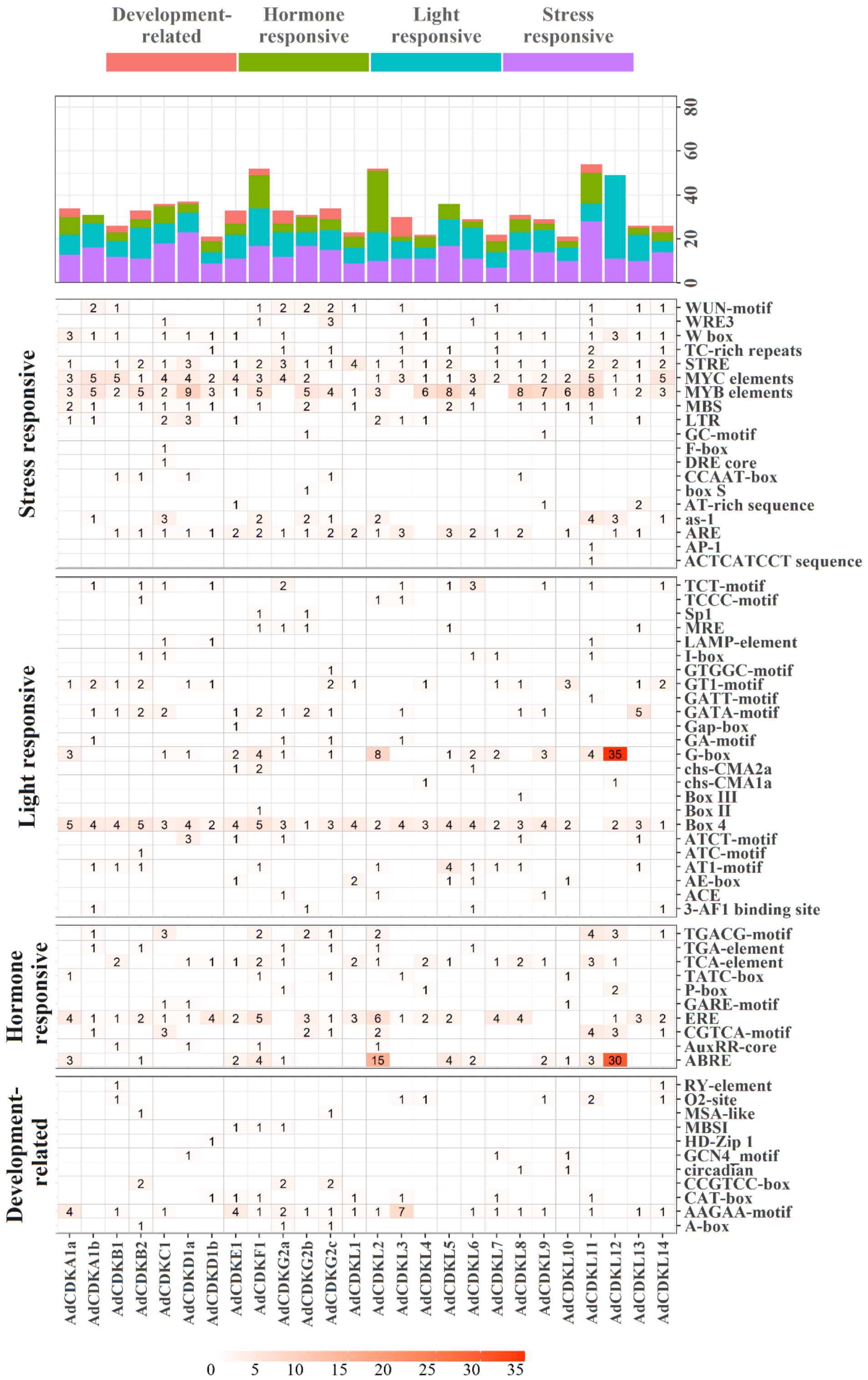

Fig. S12. The cis-elements distribution in the promoter of *CDK* and *CDKL* genes in *Arachis duranensis*. In upper panel, different colors was used to recognize various types of promoter cis-elements, like development related, phytohormone-responsive, light responsive and stress responsive. In lower panel, number of each cis-acting element of the *CDK* and *CDKL* genes promoter region (1.5 kb upstream of the translation start site).

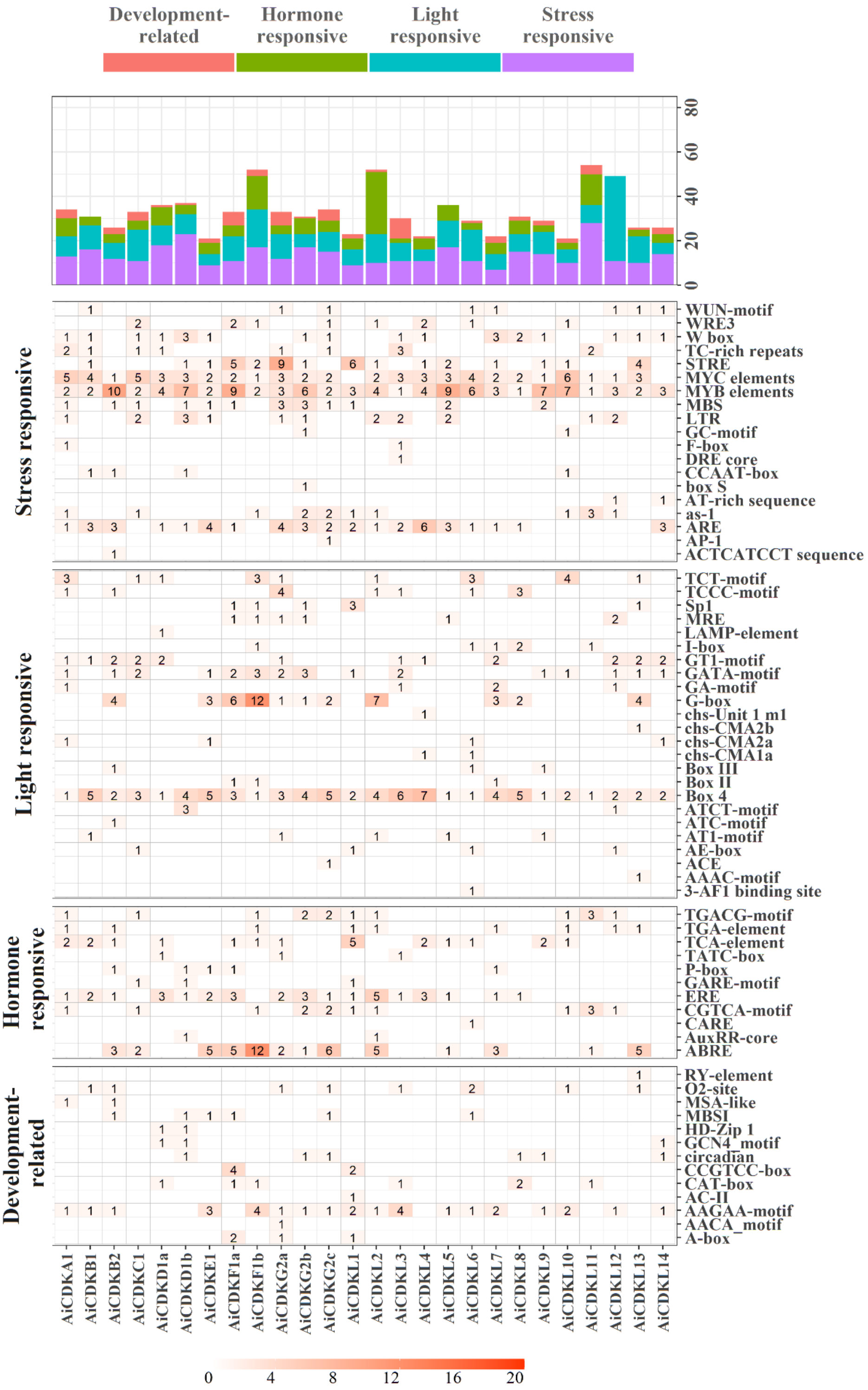

Fig. S13. The cis-elements distribution in the promoter of *CDK* and *CDKL* genes in *Arachis ipaensis*. In upper panel, different colors was used to recognize various types of promoter cis-elements, like development related, phytohormone-responsive, light responsive and stress responsive. In lower panel, number of each cis-acting element of the CDK and CDKL gene promoter region (1.5 kb upstream of the translation start site).

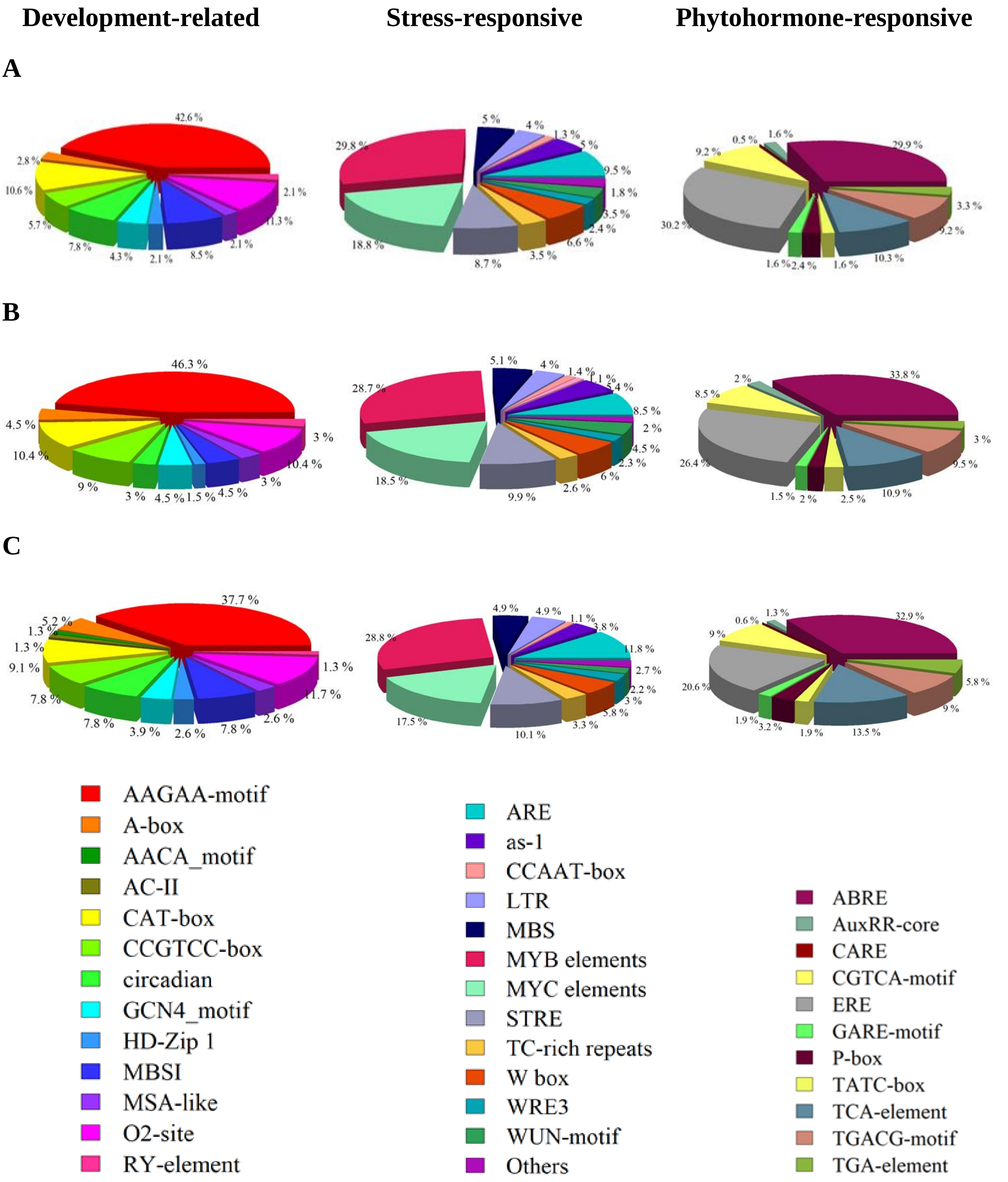

Fig. S14. Percentage of promoter cis-elements in cultivated peanut and its diploid parents. (A) *Arachis hypogaea* (B) *Arachis duranensis* and (C) *Arachis ipaensis*.
